# Exploratory Temporal and Evolutionary Insights into the *Filoviridae* Family Through Multiprotein Phylogeny

**DOI:** 10.3390/microorganisms13102388

**Published:** 2025-10-17

**Authors:** Thiago S. Messias, Kaique C. P. Silva, Narciso A. Vieira, Gislaine A. Querino, Elaine C. Marcos, Mateus J. de C. Stefani, Ana P. R. Battochio, Thaís M. Oliveira, Ivan S. Vieira, Aline S. Ibanes, Taylor E. T. Olivo, Edson C. de Melo, Silvia C. Arantes, Pedro C. R. da Luz, Maria G. R. Mengoa, Simone Soares

**Affiliations:** 1 Hospital for Rehabilitation of Craniofacial Anomalies, University of São Paulo, Rua Sílvio Marchione 3-20, Bauru 17012-900, SP, Brazil; kaiquecesar@alumni.usp.br (K.C.P.S.); marchini@usp.br (T.M.O.); tayloretolivo@usp.br (T.E.T.O.); edcar.infecto@fob.usp.br (E.C.d.M.); silviacarantes@usp.br (S.C.A.); sisoares@usp.br (S.S.); 2 Faculty of Medicine, Nove de Julho University, Rua Nicolau Assis, nº 15, Bauru 17011-102, SP, Brazil; 3 Bauru Integrated Colleges, Rua José Santiago, nº 16-50, Bauru 17056-120, SP, Brazil; biomedicina@fibbauru.br; 4 Bauru School of Dentistry, University of São Paulo, Alameda Octávio Pinheiro Brisolla 9-75, Bauru 17012-901, SP, Brazil; vieirana@usp.br; 5 Lauro de Souza Lima Institute, Rodovia Comandante Joao Ribeiro de Barros km 225–226, Bauru 17034-971, SP, Brazil; gislainequerino@hotmail.com (G.A.Q.); emarcos@ilsl.br (E.C.M.); 6 Faculty of Logistics, Estácio de Sá University, R. Clara Vendramin 58—Mossunguê, Curitiba 81200-170, PR, Brazil; mateusjstefani@gmail.com; 7 Department of Pediatric Dentistry, Orthodontics and Public Health, Bauru School of Dentistry, University of São Paulo, Alameda Dr. Octávio Pinheiro Brisolla 9-75, Bauru 17012-901, SP, Brazil; 8 School of Medicine, Federal University of São Paulo, Rua Botucatu n.º 740, Vila Clementino, São Paulo 04023-062, SP, Brazil; ivan.saccon01@gmail.com; 9 Emilio Ribas Institute of Infectology, Av. Dr Arnaldo, 165, Cerqueira Cesar, São Paulo 01246-900, SP, Brazil; aline.ibanes@emilioribas.sp.gov.br; 10 Diagnósticos do Brasil (DB Pathology Unit), Av. Victor Andrew 1470, Zona Industrial, Sorocaba 18086-390, SP, Brazil; pedro.cazadei@icloud.com; 11 Department of Prosthodontics and Periodontology, Bauru School of Dentistry, University of São Paulo, Alameda Octávio Pinheiro Brisolla 9-75, Bauru 17012-901, SP, Brazil; magabrielarobles@usp.br

**Keywords:** filoviridae, ebolavirus, marburgvirus, phylogeny, biological evolution

## Abstract

Filoviruses are among the most lethal viral human pathogens known, with significant relevance to public health, yet their evolutionary history remains poorly resolved. This study applied a multiprotein molecular phylogenetic approach to investigate the evolutionary and temporal dynamics of the family *Filoviridae*. Amino acid sequences from the proteome and seven individual proteins (NP, VP35, VP40, GP, VP30, VP24, L) were analyzed using MEGA 12, with RelTime inference anchored on uniform calibrations, and integration of epidemiological data (cases, fatalities, case fatality). The phylogenetic reconstructions revealed robust topologies for most proteins, though selective pressures on GP, VP30 and VP40 generated more variable patterns. Temporal inferences supported the classification of filoviruses into three groups: an ancestral lineage (>1 MYA, fish- and reptile-associated), an intermediate lineage (BCE–1 MYA, bat-associated), and a contemporary lineage (CE, ebolaviruses and marburgviruses). VP30 and VP40 showed consistent associations with epidemiological outcomes in *Orthoebolavirus zairense*, suggesting their interplay may underlie enhanced dispersal and virulence. Contrariwise, *Orthoebolavirus restonense* emerged as a natural counterpoint for comparison with other potential human pathogenic filoviruses. Taken together, these findings highlight that filoviral evolution is intrinsically linked not only to viral biology but also to the ecology and history of their hosts.

## 1. Introduction

The *Filoviridae* family comprises enveloped viruses with filamentous morphology and a non-segmented, negative-sense single-stranded RNA (ssRNA−) genome. Their genes are typically organized in the order 3′-*NP*-*VP35*-*VP40*-*GP*-*VP30*-*VP24*-*L*-5′, totaling approximately 19 kilobases. Members of this family are epidemiologically associated with outbreaks of high lethality, which characterizes them as emerging and reemerging pathogens of public health importance [1].

Marburgviruses (genus *Orthomarburgvirus*) were the first filoviruses to be described, in 1967, in Germany, originating from non-human primates imported from Uganda [2]. Since then, outbreaks and sporadic cases have been reported, associated with hemorrhagic fevers in sub-Saharan Africa [1].

The genus *Orthoebolavirus* comprises six species, of which *Orthoebolavirus zairense*, *Orthoebolavirus sudanense*, and *Orthoebolavirus bundibugyoense* are associated with high dissemination and pathogenicity in humans and non-human primates [3]. *Orthoebolavirus restonense*, in turn, poses a risk to non-human primates and can also infect pigs, being considered relevant to veterinary public health due to its circulation in Southeast Asia [3,4].

New members of the family have been identified through metagenomics, including cuevaviruses (bat—Spain) [5], dianloviruses (bat—China) [6], loeboviruses (fish—Switzerland) [3], oblaviruses (fish—Switzerland) [7], striaviruses (toadfish—China) [8], tapjoviruses (snake—Brazil) [9], and thamnoviruses (fish—Switzerland) [8]. These discoveries highlight the broad diversity and dynamic distribution of filoviruses [3].

Given their public health relevance and diversity [1], this study adopted an integrated multiprotein molecular phylogenetic approach to investigate the *Filoviridae* family. Amino acid sequences from the complete proteome and individual proteins (NP, VP35, VP40, GP, VP30, VP24, and L) were analyzed with three main objectives: (i) to reconstruct phylogenetic relationships; (ii) to infer ancestral nodes and estimate approximate speciation dates through temporal analyses; (iii) to assess correlations between phylogenetic structure and epidemiological data (human cases, deaths, and case fatality). This integration aims to broaden the understanding of the evolutionary dynamics of filoviruses, while reducing single-protein bias and capturing complementary evolutionary signals that provide a more robust basis for linking phylogeny with epidemiological outcomes. Together, these objectives address both evolutionary reconstruction and epidemiological correlation, highlighting the translational potential of filovirus phylogeny for public health.

## 2. Materials and Methods

The experiments were organized into phases corresponding to the proteome and the proteins NP, VP35, VP40, GP, VP30, VP24, and L. The sampling group was defined based on the taxonomic references of the International Committee on Taxonomy of Viruses (ICTV) [3,10]. Amino acid sequences, when available, were retrieved from NCBI Virus (National Library of Medicine, Bethesda, MD/USA) [11]. Phylogenetic analyses were conducted using the MEGA version 12 software [12], with full methodological details provided in File S1: Phylogenetic Reconstruction. Epidemiological data (number of cases, deaths, and case fatality), when available, were extracted from the Centers for Disease Control and Prevention (Atlanta, GA/USA) [13,14] and the textbook Fields Virology: Emerging Viruses [1].

The sample set included the following viral species, with respective abbreviations and number of sequences: *Cuevavirus lloviuense* (LLOV/1); *Dianlovirus menglaense* (MLAV/1); *Oblavirus percae* (OBLV/1); *Orthoebolavirus bombaliense* (BOMV/1); *Orthoebolavirus bundibugyoense* (BDBV/2); *Orthoebolavirus restonense* (RESV/5); *Orthoebolavirus sudanense* (SUDV/6); *Orthoebolavirus taiense* (TAIV/1); *Orthoebolavirus zairense* (EBOV/19); *Orthomarburgvirus marburgense* (MARV/12); *Striavirus antennarii* (XILV/1); *Tapjovirus bothropis* (TAPV/1); *Thamnovirus kanderense* (KANV/1); *Thamnovirus percae* (FIWV/1) and *Thamnovirus thamnaconi* (HUJV/1). The species *Dianlovirus dehongense* and *Loebovirus percae* were excluded due to the unavailability of sequences. In addition, for VP24, VP30, VP35, and VP40 phases, some species (e.g., XILV, KANV, FIWV, HUJV, OBLV) were excluded because specific protein sequences were naturally absent.

The species *Lyssavirus rabies* (RABV) was selected as the outgroup, given its evolutionary and taxonomic position closely related at the order level but distinct at the family level. Specifically, RABV belongs to the family *Rhabdoviridae*, which, together with the family *Filoviridae*, comprises the order *Mononegavirales* [10].

Regarding sample organization, sequence identifications are provided in Table S1: Experimental phases, followed by their respective strain IDs, formatted as follows: abbreviation corresponding to the viral species/last two digits of the year of isolation or identification/first three letters or abbreviation of the location of origin. Example: TAPV18Bra, corresponding to *Tapjovirus bothropis*, identified in 2018 from a sample collected in Brazil. The columns Abbreviation/Species, Location, Year of Identification, Number of Human Cases, Number of Human Deaths, and Case Fatality present the corresponding data. The Bootstrap I column provides replicate values that indicate the topological statistical significance of each sample ID. The Cluster (groups of viruses forming supported clades in the phylogenetic reconstructions)/Singleton (individual viral taxa that did not cluster with other sequences but were retained to preserve representativeness of rare or unique isolates) column groups the respective sample IDs; cluster nomenclature is based on ancestral inference results, using the oldest sample within the group (e.g., EBOV1976, in which the oldest ID is EBOV76DRC). When a species forms a single cluster or remains isolated (singleton), the cluster name corresponds to the species (e.g., SUDV, where all IDs of *Orthoebolavirus sudanense* formed a single cluster). When the cluster is shared between species, it is indicated by a slash (e.g., TAIV/BDBV). An exception is the cluster FishFilo, which groups filoviruses identified in fish. The final column presents Bootstrap II values corresponding to the ancestor that originated each cluster/singleton. In the Proteome phase (Table S1: Experimental phases, Proteome spreadsheet), an additional column (Approximate Temporal Analysis) is included, referring to the temporal analysis of speciation, with dates expressed in BCE (Before Common Era), CE (Common Era), and MYA (millions of years ago). In this context, the term lineage refers specifically to the temporal framework, denoting broader categories of divergence timing.

To evaluate statistically significant differences, the clusters/singletons obtained from phylogenetic reconstructions were organized according to three epidemiological variables: number of human cases (sum), number of human deaths (sum), and human case fatality (mean). Normality was assessed using the Shapiro–Wilk test, followed by the nonparametric Kruskal–Wallis test. The Mann–Whitney U test was applied as a post hoc analysis, comparing each cluster/singleton with the remainder of the group. Statistical significance was corrected by FDR (Benjamini–Hochberg, α = 0.05), using ChatGPT, version GPT-5 (OpenAI, San Francisco, CA, USA) [15], and SPSS Statistics 28.0 software (IBM, North Castle, NY, USA) [16].

## 3. Results

### 3.1. Proteome Phase (Table S1: Experimental Phases, Proteome Spreadsheet)

The best-fit evolutionary model was identified as the Le and Gascuel (LG) with empirical frequencies (+F) and gamma distribution (+G). The results were: BIC (Bayesian Information Criterion): 122,424.394; AICc (Corrected Akaike Information Criterion): 121,189.000 and lnL (Log-Likelihood): −60,467.369.

**Figure 1:**
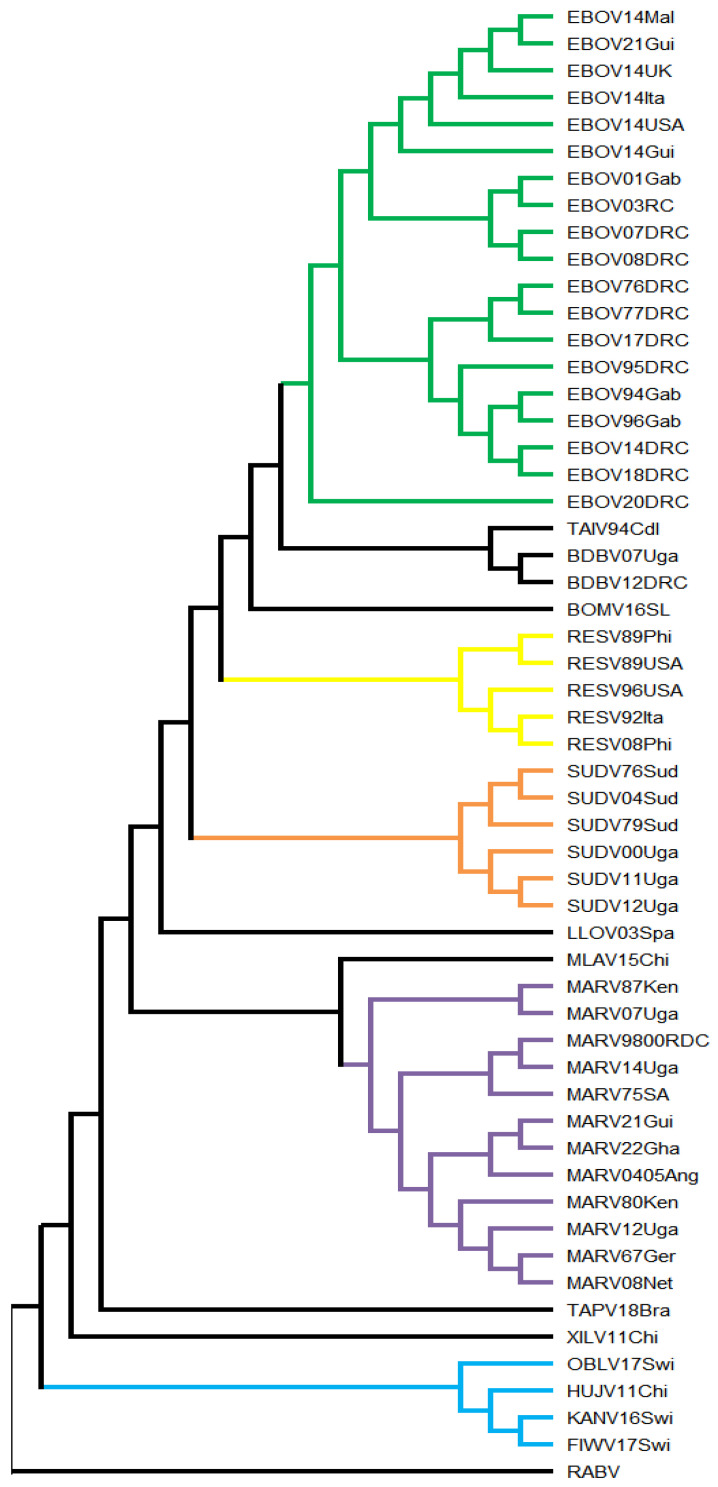
Filoviral Phylogenetic Tree (Proteome). *Orthoebolavirus zairense* (EBOV) cluster in green, *Orthoebolavirus restonense* (RESV) cluster in yellow, *Orthoebolavirus sudanense* (SUDV) cluster in orange, *Orthomarburgvirus marburgense* (MARV) cluster in purple, FishFilo cluster in blue. Taxon IDs: MARV67Ger—MARV Germany 1967; MARV75SA—MARV South Africa 1975; SUDV76Sud—SUDV Sudan 1976; EBOV76DRC—EBOV Democratic Republic of the Congo 1976; EBOV77DRC—EBOV Democratic Republic of the Congo 1977; SUDV79Sud—SUDV Sudan 1979; MARV80Ken—MARV Kenya 1980; MARV87Ken—MARV Kenya 1987; RESV89Phi—RESV Philippines 1989; RESV89USA—RESV United States of America 1989; RESV92Ita—RESV Italy 1992; TAIV94CdI—*Orthoebolavirus taiense* (TAIV) Côte d’Ivoire 1994; EBOV94Gab—EBOV Gabon 1994; EBOV95DRC—EBOV Democratic Republic of the Congo 1995; RESV96USA—RESV United States of America 1996; EBOV96Gab—EBOV Gabon 1996; MARV9800DRC—MARV Democratic Republic of the Congo 1998–2000; SUDV00Uga—SUDV Uganda 2000; EBOV01Gab—EBOV Gabon 2001; EBOV03RC—EBOV Republic of the Congo 2003; MARV0405Ang—MARV Angola 2004–2005; SUDV04Sud—SUDV Sudan 2004; MARV07Uga—MARV Uganda 2007; BDBV07Uga—*Orthoebolavirus bundibugyoense* (BDBV) Uganda 2007; EBOV07DRC—EBOV Democratic Republic of the Congo 2007; MARV08Net—MARV Netherlands 2008; EBOV08DRC—EBOV Democratic Republic of the Congo 2008; RESV08Phi—RESV Philippines 2008; SUDV11Uga—SUDV Uganda 2011; MARV12Uga—MARV Uganda 2012; SUDV12Uga—SUDV Uganda 2012; BDBV12DRC—BDBV Democratic Republic of the Congo 2012; MARV14Uga—MARV Uganda 2014; EBOV14DRC—EBOV Democratic Republic of the Congo 2014; EBOV14Gui—EBOV Guinea 2014; EBOV14Ita—EBOV Italy 2014; EBOV14Mal—EBOV Mali 2014; EBOV14UK—EBOV United Kingdom 2014; EBOV14USA—EBOV United States of America 2014; EBOV17DRC—EBOV Democratic Republic of the Congo 2017; EBOV18DRC—EBOV Democratic Republic of the Congo 2018; EBOV20DRC—EBOV Democratic Republic of the Congo 2020; MARV21Gui—MARV Guinea 2021; EBOV21Gui—EBOV Guinea 2021; MARV22Gha—MARV Ghana 2022; LLOV03Spa—*Cuevavirus lloviuense* (LLOV) Spain 2003; MLAV15Chi—*Dianlovirus menglaense* (MLAV) China 2015; BOMV16SL—*Orthoebolavirus bombaliense* (BOMV) Sierra Leone 2016; TAPV18Bra—*Tapjovirus bothropis* (TAPV) Brazil 2018; OBLV17Swi—*Oblavirus percae* (OBLV) Switzerland 2017; KANV16Swi—*Thamnovirus kanderense* (KANV) Switzerland 2016; XILV11Chi—*Striavirus antennarii* (XILV) China 2011; FIWV17Swi—*Thamnovirus percae* (FIWV) Switzerland 2017; HUJV11Chi—*Thamnovirus thamnaconi* (HUJV) China 2011; RABV—*Lyssavirus rabies*.

The phylogenetic tree corresponding to the filovirus proteomes is shown in Figure 1.

No Bootstrap values below 70 were observed, indicating a robust evolutionary reconstruction of filovirus proteome sequences. EBOV, RESV, SUDV and MARV formed well-defined clusters.

Within the EBOV cluster, four subclusters were formed according to isolation dates and locations, except for EBOV21Gui, which was grouped within the 2014 isolates. In addition, the EBOV20DRC isolate suggests descent from the ancestral node of the remaining EBOV sequences.

TAIV and BDBV share the same filoviral ancestor, while BOMV shares ancestry with EBOV, TAIV, and BDBV.

RESV (yellow) and SUDV (orange) formed clusters consistent with expectations, based on their isolation dates.

The ancestral node of LLOV separates the genera *Orthoebolavirus* (EBOV, TAIV, BDBV, BOMV, RESV and SUDV) and *Cuevavirus* (LLOV).

MARV (purple) exhibited clear evolutionary diversity, particularly between the cluster formed by MARV87Ken and MARV07Uga compared with the remaining sequences. The ancestral node of MARV diverges toward MLAV, separating the genera *Orthomarburgvirus* and *Dianlovirus*.

The genus *Tapjovirus* (TAPV) shares the same ancestor as the aforementioned genera, which in turn shares filoviral ancestry with the genus *Striavirus* (XILV).

At the base of the tree, based on currently available sequences, these genera share a putative ancestral filovirus with the FishFilo cluster, which comprises the genera *Oblavirus* (OBLV) and *Thamnovirus* (HUJV, FIWV and KANV).

Based on the generated topology, 20 clusters/singletons were defined, and none of their ancestral nodes showed Bootstrap values below 70.

The molecular clock test rejected the null hypothesis (*p* = 6.148 × 10^−15^), indicating that the sequences analyzed in the Proteome phase display variable evolutionary rates across lineages. Results from the RelTime analysis are presented in Table 1.

Regarding the clusters/singletons formed and their statistical outcomes, the Shapiro–Wilk normality test indicated a non-normal distribution (*p* < 0.05). Subsequently, the Kruskal–Wallis test revealed significant results for the categories: number of human cases (*p* = 0.019), number of human deaths (*p* = 0.010), and case fatality (*p* = 0.006). In the post hoc Mann–Whitney U test with FDR correction (Benjamini–Hochberg, α = 0.05), the following clusters/singletons showed statistically significant results:

Number of cases—EBOV1994 (*p* = 0.009), EBOV2001 (*p* = 0.048), and FishFilo (*p* = 0.004);

Number of deaths—RESV1992 (*p* = 0.034), EBOV1994 (*p* = 0.006), EBOV2001 (*p* = 0.033), and FishFilo (*p* = 0.013);

Case fatality—RESV1992 (*p* = 0.035), MARV2004 (*p* = 0.036), and FishFilo (*p* = 0.014).

### 3.2. GP Phase (Table S1: Experimental Phases, GP Spreadsheet)

The best-fit evolutionary model was identified as the Jones–Taylor–Thornton (JTT) with gamma distribution (+G). The results were: BIC: 11,673.367; AICc: 10,935.953 and lnL: −5358.278.

The phylogenetic tree corresponding to the filovirus glycoproteins (GP) is shown in Figure S1: Filoviral Phylogenetic Tree (GP).

Bootstrap values below 70 were observed, suggesting that certain positions in the tree may not represent a robust evolutionary scenario. Therefore, only results with Bootstrap values equal to or above 70 will be considered hereafter. MARV, RESV, SUDV, FishFilo and TAIV/BDBV formed well-defined clusters.

TAIV and BDBV share the same ancestral GP, which in turn shares ancestry with the GP that diverged into SUDV and RESV.

MARV and MLAV share ancestry in GP formation.

Based on the generated topology, 16 clusters/singletons were formed. Their statistical results showed that the Shapiro–Wilk test indicated a non-normal distribution (*p* < 0.05), followed by the Kruskal–Wallis test, which yielded significant results for the categories: number of human cases (*p* = 0.00924), number of human deaths (*p* = 0.00527), and human case fatality (*p* = 0.00381). In the post hoc Mann–Whitney U test with FDR correction (Benjamini–Hochberg, α = 0.05), the following clusters/singletons showed statistically significant results:

Number of deaths—RESV (*p* = 0.005), EBOV1994 (*p* = 0.006), and FishFilo (*p* = 0.013).

### 3.3. L Phase (Table S1: Experimental Phases, L Spreadsheet)

The best-fit evolutionary model was identified as the LG+F+G. The results were: BIC: 81,756.121; AICc: 80,563.949 and lnL: −40,154.790.

The phylogenetic tree corresponding to filovirus L (Large) proteins is shown in Figure S2: Filoviral Phylogenetic Tree (L).

A Bootstrap value below 70 was observed in the tree topology, specifically at the ancestral node of the XILV11Chi singleton (69). All other nodes and branches showed robust phylogenetic reconstruction.

The phylogenetic reconstruction of the L protein yielded a topology similar to that observed in the proteome tree (Figure 1).

Based on the generated topology, 22 clusters/singletons were defined. Statistical analysis indicated a non-normal distribution by the Shapiro–Wilk test (*p* < 0.05), followed by the Kruskal–Wallis test, which revealed significant results for the categories: number of human cases (*p* = 0.027), number of human deaths (*p* = 0.016), and human case fatality (*p* = 0.009). In the post hoc comparisons (Mann–Whitney U), *p*-values < 0.05 were observed; however, after FDR correction (Benjamini–Hochberg, α = 0.05), no results remained significant.

### 3.4. NP Phase (Table S1: Experimental Phases, NP Spreadsheet)

The best-fit evolutionary model was identified as the LG+G. The results were: BIC: 15,901.167; AICc: 15,066.923 and lnL: −7424.762.

The phylogenetic tree corresponding to filovirus nucleoproteins (NP) is shown in Figure S3: Filoviral Phylogenetic Tree (NP).

Two Bootstrap values below 70 were observed in the tree topology, specifically at the ancestral node of MARV75SA and MARV80Ken (53), and at their ancestral node (51). All other nodes and branches showed robust phylogenetic reconstruction.

The phylogenetic reconstruction of the nucleoprotein yielded a topology similar to that observed in the proteome (Figure 1) and L protein (Figure S2) trees. However, in this reconstruction, TAPV resumed the position inferred in the proteome-based analysis, i.e., sharing ancestry with the ancestral mammalian filovirus.

SUDV was positioned at an earlier node compared with its placement in the L (Figure S2) and proteome (Figure 1) phases, indicating that its nucleoproteins speciated at the ancestral node of the remaining *Orthoebolavirus* members.

BOMV diverged from the ancestor of EBOV, RESV, TAIV, and BDBV, differing from the proteome tree (Figure 1), where it originated from the same ancestral node as EBOV, TAIV, and BDBV.

Based on the generated topology, 19 clusters/singletons were formed. Statistical analyses indicated a non-normal distribution by the Shapiro–Wilk test (*p* < 0.05), followed by the Kruskal–Wallis test, which yielded significant results for the categories: number of human cases (*p* = 0.02257), number of human deaths (*p* = 0.00973), and human case fatality (*p* = 0.00566). In the post hoc Mann–Whitney U comparisons, FishFilo, EBOV1976, and RESV clusters stood out with *p* < 0.05 in multiple outcomes. However, after FDR correction (α = 0.05), none remained significant.

### 3.5. VP24 Phase (Table S1: Experimental Phases, VP24 Spreadsheet)

The best-fit evolutionary model was identified as the LG+G. The results were: BIC: 9190.018; AICc: 8468.422 and lnL: −4135.385.

The phylogenetic tree corresponding to filovirus protein 24 (VP24) is shown in Figure S4: Filoviral Phylogenetic Tree (VP24).

Bootstrap values below 70 were observed in the tree topology, rendering the position of certain clusters uncertain, including MARV1967 (69), SUDV1976 (67), SUDV2000 (69), EBOV2001 (57), EBOV2007 (66) and EBOV1976 (68). All other nodes showed robust phylogenetic reconstruction.

The phylogenetic reconstruction of VP24 yielded a topology similar to that observed in the proteome (Figure 1), L protein (Figure S2) and nucleoprotein (Figure S3) trees. However, in this reconstruction, the EBOV1994 cluster was formed (EBOV14DRC, EBOV14USA, EBOV14Gui, EBOV18DRC, EBOV96Gab, EBOV14Mal, EBOV94Gab, EBOV14UK, EBOV14Ita, EBOV17DRC, EBOV21Gui, EBOV95DRC), grouping EBOV strains in a manner distinct from other reconstructed topologies.

Based on the generated topology, 15 clusters/singletons were formed. Statistical analysis indicated a non-normal distribution by the Shapiro–Wilk test (*p* < 0.05), followed by the Kruskal–Wallis test, which yielded significant results for the categories: number of human deaths (*p* = 0.03885) and human case fatality (*p* = 0.01540). In the post hoc Mann–Whitney U test with FDR correction (Benjamini–Hochberg, α = 0.05), the following clusters/singletons showed statistically significant results:

Number of deaths—RESV (*p* = 0.0026);

Case fatality—RESV (*p* = 0.0027).

### 3.6. VP30 Phase (Table S1: Experimental Phases, VP30 Spreadsheet)

The best-fit evolutionary model was identified as the LG+G. The results were: BIC: 11,360.259; AICc: 10,620.727 and lnL: −5206.107.

The phylogenetic tree corresponding to filovirus protein 30 (VP30) is shown in Figure S5: Filoviral Phylogenetic Tree (VP30).

Bootstrap values below 70 were observed in the tree topology, leaving the position of some ancestral clusters uncertain, such as the node (50) comprising XILV, TAPV, MLAV, and MARV, as well as the ancestral node (51) of RESV, BOMV, BDBV, TAIV, and EBOV. Only the ancestral node (100) of LLOV and SUDV remained robust in evolutionary terms.

Considering the speciation events closest to the division of filovirus genera, greater topographic robustness was observed, along with the formation of a single cluster grouping all EBOV strains.

Based on the generated topology, 13 clusters/singletons were defined. Statistical analysis indicated a non-normal distribution by the Shapiro–Wilk test (*p* < 0.05), followed by the Kruskal–Wallis test, which yielded significant results for the categories: number of human cases (*p* = 0.03639), number of human deaths (*p* = 0.01640), and case fatality (*p* = 0.01591). In the post hoc Mann–Whitney U test with FDR correction (Benjamini–Hochberg, α = 0.05), the following clusters/singletons showed statistically significant results:

Number of cases—EBOV (*p* = 0.0028);

Number of deaths—RESV (*p* = 0.0026), EBOV (*p* = 0.0020);

Case fatality (%)—RESV (*p* = 0.0027).

### 3.7. VP35 Phase (Table S1: Experimental Phases, VP35 Spreadsheet)

The best-fit evolutionary model was identified as the LG+G. The results were: BIC: 11,896.493; AICc: 11,154.813 and lnL: –5478.733.

The phylogenetic tree corresponding to filovirus protein 35 (VP35) is shown in Figure S6: Filoviral Phylogenetic Tree (VP35).

Bootstrap values below 70 were observed in the tree topology, specifically for the EBOV2007 (62) and MARV1975 (57) clusters. At all other points, the reconstruction demonstrated evolutionary robustness, with a topology more closely resembling the proteome, NP, and L phases.

Based on the generated topology, 16 clusters/singletons were defined. Statistical analysis indicated a non-normal distribution by the Shapiro–Wilk test (*p* < 0.05), followed by the Kruskal–Wallis test, which yielded significant results for the categories: number of human deaths (*p* = 0.02441) and case fatality (*p* = 0.01036). In the post hoc Mann–Whitney U test with FDR correction (Benjamini–Hochberg, α = 0.05), only the EBOV1976 cluster showed significance for the number of deaths (*p* = 0.0025).

### 3.8. VP40 Phase (Table S1: Experimental Phases, VP40 Spreadsheet)

The best-fit evolutionary model was identified as the LG+G. The results were: BIC: 9348.536; AICc: 8622.546 and lnL: −4210.321.

The phylogenetic tree corresponding to filovirus protein 40 (VP40) is shown in Figure S7: Filoviral Phylogenetic Tree (VP40).

Bootstrap values below 70 were observed in the tree topology at internal nodes of MARV (61), RESV (67), SUDV (55), and EBOV (66–60). At all other points, the reconstruction demonstrated evolutionary robustness, with a topology more similar to that of the VP30 phase, although with divergences not observed in other phases, such as:

Division of MARV (100) into a singleton (MARV67Ger) and a cluster comprising the remaining members;

Division of SUDV (100) into a singleton (SUDV11Uga) and a cluster comprising the remaining members;

Grouping of EBOV (100) into a large cluster, as also observed in the VP30 phase.

Based on the generated topology, 11 clusters/singletons were formed. Statistical analysis indicated a non-normal distribution by the Shapiro–Wilk test (*p* < 0.05), followed by the Kruskal–Wallis test, which yielded significant results for the categories: number of human cases (*p* = 0.00681), number of human deaths (*p* = 0.00458), and human case fatality (*p* = 0.00175). In the post hoc Mann–Whitney U test with FDR correction (Benjamini–Hochberg, α = 0.05), the following clusters/singletons showed statistically significant results:

Number of cases—EBOV (*p* = 0.0305);

Number of deaths—RESV (*p* = 0.0142), EBOV (*p* = 0.0142);

Case fatality—RESV (*p* = 0.0293).

## 4. Discussion

### 4.1. Proteome Phase

The Le and Gascuel (2008) model represents an improved amino acid substitution matrix capable of capturing complex evolutionary patterns. The gamma distribution parameter (+G) is particularly relevant for sequences with both conserved and highly variable regions [17], as commonly observed in viral proteins [18].

The Adaptive Bootstrap method, based on Felsenstein (1985) [19], yielded values above 70 in the topological reconstruction of the tree. According to Hillis and Bull (1993) [20], this result supports a reliable phylogenetic topology.

The cluster composed of members of *Orthoebolavirus zairense* (EBOV, in green, Figure 1) showed a subcluster (EBOV14Mal, EBOV14UK, EBOV14Ita, EBOV14USA, and EBOV14Gui) associated with the 2013–2016 West African outbreak (approximately 28,500 cases and 11,300 deaths; 42% case fatality) [1]. Notably, the presence of EBOV21Gui in this cluster suggests that viral descendants of this outbreak may still be circulating in West Africa. This strain was linked to a rural outbreak (23 cases, 12 deaths and 52.2% case fatality), and the CDC (2024) [13] proposed a potential association with filoviral persistence in survivors of the 2013–2016 outbreak, a hypothesis corroborated by its phylogenetic position. The placement of EBOV14DRC suggests an independent event, not derived from the major 2013–2016 outbreak. This interpretation is supported by the CDC (2024) [13], which associates this strain with the 1995 Kikwit outbreak. EBOV95DRC, also linked to this outbreak, was grouped within the same subcluster as EBOV14DRC, reinforcing this finding. Furthermore, EBOV20DRC emerged as a descendant of the common ancestor of the remaining EBOV strains. This strain, associated with 130 cases and 55 deaths (42.3% case fatality), has been suggested to represent a novel zoonotic spillover event [13], which is consistent with its phylogenetic placement.

The species *Orthoebolavirus taiense* (TAIV) was first identified following an outbreak in chimpanzees (Primate: Hominidae: *Pan troglodytes verus* Schwarz, 1934) at Taï National Park, Côte d’Ivoire, in which 43 animals either died or disappeared. Since then, only one confirmed human infection has been reported [13,21,22]. TAIV clustered with *Orthoebolavirus bundibugyoense* (BDBV), which was discovered in Uganda during a 2007 outbreak that resulted in 131 infections and 42 deaths (32% case fatality) [13,23]. In the case of BDBV, even after its reemergence in 2012, no association with non-human animal outbreaks or geographical factors was identified [1,13]. Considering that BDBV shares the same ancestral node with TAIV, it is plausible that its natural and/or intermediate reservoir may also be related to non-human primates.

BOMV16SL, which shares the common ancestor of EBOV, TAIV, and BDBV, belongs to *Orthoebolavirus bombaliense* (BOMV). It was discovered through Next-Generation Sequencing (NGS) in bats (Chiroptera: Molossidae: *Chaerophon pumilus* Cretzschmar, 1826), reinforcing the role of bats as reservoirs of the genus *Orthoebolavirus* [24].

*Orthoebolavirus restonense* (RESV, yellow, Figure 1) formed two subclusters, supporting its historical exportation events from the Philippines to the United States in 1989 (RESV89Phi and RESV89USA), and to Italy and the United States in the 1990s (RESV96USA, RESV92Ita, and RESV08Phi) [13]. However, phylogenetic inference suggests that these exported strains likely originated from distinct lineages, as RESV92Ita and RESV08Phi share the same ancestor, which in turn is also related to the strain exported to the United States in 1996 (RESV96USA). Within the genus *Orthoebolavirus*, RESV displays the broadest host range. The exportation events mentioned above occurred in non-human primates (Primates: Cercopithecidae: *Macaca fascicularis* Raffles, 1821) [13,25,26]. Additionally, RESV has been detected in pigs (Artiodactyla: Suidae: *Sus scrofa domesticus* Erxleben, 1777) [27] and in bats, through the identification of genetic fragments [28]. To date, no association with human disease has been documented, although the possibility of infection remains [13,29].

LLOV, represented by the species *Cuevavirus lloviuense*, was first identified in Spain in 2003, in the host Schreibers’ long-fingered bat (Chiroptera: Miniopteridae: *Miniopterus schreibersii* Kuhl, 1817) [5]. Its phylogenetic position, sharing an ancestor with the genus *Orthoebolavirus*, corroborates the findings of Biedenkopf et al. (2024) [3].

The species *Dianlovirus menglaense*, represented by strain MLAV15Chi, was discovered in 2015 in China in bats of the genus *Rousettus* [6,30]. This species shares the same ancestor as MARV, which in turn has natural hosts in bats of the genus *Rousettus*, specifically Chiroptera: Pteropodidae: *Rousettus aegyptiacus* E. Geoffroy, 1810 [31].

Regarding the species *Orthomarburgvirus marburgense*, the clustering pattern reflected dispersal dynamics consistent with its epidemiological history [1,14]. Four clusters were identified: The first comprised MARV87Ken and MARV07Uga. MARV87Ken was isolated from a single fatal case of a boy during a vacation trip to Kenya in 1987 and became known as the Ravn virus [1,14,32]. MARV07Uga was isolated from a 2007 outbreak (4 cases, 1 death and 25% case fatality) linked to visits to a gold mine in Uganda, and was also classified as a Ravn virus strain [1]. The second cluster (MARV9800DRC, MARV14Uga, and MARV75SA), the third (MARV21Gui, MARV22Gha, and MARV0405Ang), and the fourth (MARV80Ken, MARV12Uga, MARV67Ger, and MARV08Net) are related to Marburg virus variants and clearly share ancestry with the Ravn virus cluster, corroborating the phylogenetic analyses of Biedenkopf et al. (2024) [3].

Distant from the aforementioned filoviruses but sharing the same ancestor lies the species *Tapjovirus bothropis* (TAPV). To date, TAPV is the only filovirus species identified in reptiles, specifically in the northern jararaca (Serpentes: Viperidae: *Bothrops atrox* Linnaeus, 1758), native to Brazil. The fact that TAPV shares ancestry with mammalian filoviruses corroborates its genomic organization, which is similar to that of mammalian filoviruses, although phylogenetically it remains isolated and more closely related to fish filoviruses [3,9].

XILV, belonging to the species *Striavirus antennarii*, was isolated from the spotted frogfish in the East China Sea (Lophiiformes: Antennariidae: *Antennarius striatus* Shaw, 1794) through NGS. Preceding XILV is the FishFilo cluster, composed of fish-infecting filoviruses: OBLV, of the species *Oblavirus percae*, isolated from the European perch (Perciformes: Percidae: *Perca fluviatilis* Linnaeus, 1758); HUJV, of the species *Thamnovirus thamnaconi*; KANV, of the species *Thamnovirus kanderense* and FIWV, of the species *Thamnovirus percae*. In addition to being identified in the European perch, *Thamnovirus* has also been detected in the greenfin horse-faced filefish (Tetraodontiformes: Monacanthidae: *Thamnaconus septentrionalis* Günther, 1874) [7,8].

Prior to temporal inference, a molecular clock test must be performed to determine whether the evolutionary rates of the analyzed sequences are constant or variable. This assessment guides the choice between strict or relaxed temporal inference methodologies [33]. Considering that the analyzed sequences derive from viruses with ribonucleic acid genomes, the molecular clock test indicating variable evolutionary rates across lineages corroborates the high mutational rates of these viruses, particularly when compared with viruses that possess deoxyribonucleic acid genomes [18,34].

Temporal inference was anchored using two uniform calibration points. At the ancestral node of filoviruses, the emergence was estimated to fall between 28 and 400 million years ago [35]. Further down the tree, specifically at the divergence node between the genera *Orthoebolavirus* and *Orthomarburgvirus*, the estimates of Suzuki & Gojobori (1997) were applied, proposing that ebolaviruses and marburgviruses diverged approximately 7100 to 7900 years ago [36].

Temporal inference results indicate that the ancestor of the FishFilo cluster (OBLV17Swi, FIWV17Swi, HUJV11Chi, and KANV16Swi) likely emerged around 32 MYA. Geologically, this age corresponds to the Cenozoic Era, Tertiary Period (Paleogene), Oligocene Epoch, Rupelian Age (27.82–33.9 MYA) [37]. During this time, Earth was undergoing a gradual cooling trend following the warmth of the Eocene. Polar ice sheets began forming in Antarctica, leading to lower sea levels and more seasonal climates [38]. The climatic complexity of this period also suggests that eastern China experienced relatively humid conditions, influenced by proximity to the Pacific Ocean. Geological formations such as the Huagang in the Xihu Sag (East China Sea), dated to the Oligocene, reveal a transitional sedimentary environment between marine and terrestrial settings, with evidence of deltas, shallow lagoons, and fluviomarine deposition [39]. When considering the hosts in which FishFilo viruses have been identified, the inferred age does not align perfectly. The species *Perca fluviatilis*, distributed mainly across Eurasia, is estimated to have originated around 19.8 MYA, during the Miocene [40]. However, if we adopt the estimate of Collette & Bănărescu (1977) [41], which places the origin of the Percidae family at 58–68 MYA in the Paleocene, the inferred time of filovirus emergence becomes consistent with the diversification of its hosts. Further investigation of filoviruses in other fish genera could help resolve this issue.

XILV11Chi was associated with an estimated ancestral emergence time of approximately 11 MYA, placing this filoviral lineage in the Neogene Period, Miocene Epoch, between the Serravallian (11.63–13.82 MYA) and Tortonian (7.246–11.63 MYA) ages [37]. During this epoch, Earth exhibited conditions broadly similar to the present, but with highly dynamic climates, it began with glaciation episodes and ended with a period of elevated temperatures. In parallel, flora and fauna evolved into ancestors more closely resembling modern forms [42]. Regarding its host, the frogfish, the family Antennariidae includes species distributed across all oceans and seas. These fishes can use their fins to achieve limited locomotion out of water, though in an extremely constrained capacity [43]. The inferred divergence time of the genus *Antennarius* ranges from 9.4 to 24.9 MYA, according to the findings of Muschick, Rüber, and Matschiner (2025) [44], which corroborates the inferred timing of the filoviral ancestor associated with XILV.

TAPV18Bra showed an estimated ancestral emergence of approximately 10 MYA, placing this ancestry within the Miocene Epoch, Tortonian Age (7.246–11.63 MYA) [37]. Regarding the genus *Bothrops* (the host in which TAPV was identified), its diversification likely occurred between 10 and 5 MYA [45], which is consistent with the temporal inference for TAPV. During this period, much of what is now the Amazon basin was covered by the so-called Pebas System, an immense complex of lakes, swamps, and wetlands that extended across millions of square kilometers. This aquatic system formed because the Andes were still undergoing significant uplift, blocking drainage into the Pacific Ocean and creating a massive inland basin. While tectonic plates continued to reposition continents, South America remained isolated, allowing endemic species to evolve. Fossil evidence suggests that the family Viperidae had already inhabited South America for approximately 20 MYA [45,46].

Entering the Holocene Epoch, Northgrippian Age (8200–4200 yrs) [37], temporal inference for LLOV03Spa indicates an ancestral emergence around 3433 BCE. Quantitative palynological analysis by Liu et al. (2023) shows that, during the Holocene, the west–east humidity gradient in the Iberian Peninsula was less pronounced than in modern times, suggesting a relatively homogeneous climatic distribution that supported the fertility of aquatic and fluvial ecosystems [47]. In parallel, the presence of the family Miniopteridae during the Quaternary establishes that insectivorous bats of this family already inhabited caves and Mediterranean landscapes, both coastal and inland. They fed on aquatic insects and migrated along favorable ecological corridors since the Pleistocene (2.28 MYA–12,000 yrs) [37,48]. These characteristics reinforce the plausibility of the temporal inferences obtained for LLOV.

For the species *Dianlovirus menglaense*, represented by MLAV15Chi, its ancestral lineage was estimated to have emerged around 1569 BCE, within the Meghalayan Age (4200 yrs to the present) [37]. The bat genus in which MLAV was identified is *Rousettus*, belonging to the family Pteropodidae, which comprises several Old World fruit bats with wide distribution across Asia, Africa, and Australia [49]. These bats are predominantly frugivorous [50], occupy diverse habitats, including cave-dwelling behavior [51], and exhibit dynamic adaptability to urban environments [52]. Their ecological importance is critical, particularly in seed dispersal, pollination, and forest regeneration. However, they may also serve as natural reservoirs for several potential pathogens [53]. The inferred timing and geographic context of MLAV coincide with the Bronze Age in China (Shang Dynasty, 1600–1027 BCE). Considering the possible conflicts, environmental transformations, and resource exploitation of that period [54], along with the fact that MLAV shares a common ancestor with MARV, several scenarios remain open regarding the emergence and dispersal of filoviruses during that time.

MARV, which shares ancestry with MLAV, showed estimated emergence dates between 1773 CE and 1959 CE. The strains MARV87Ken and MARV07Uga, known as the Ravn virus and sharing the same ancestor as other MARV clusters, are inferred to have speciated around 1773 CE. At this time, Uganda was undergoing profound ecological and sociocultural transformations. In 1773, the region was at the height of the Buganda Kingdom’s power, which had established itself as a dominant state, asserting increasing supremacy over lacustrine areas and neighboring territories such as Bunyoro-Kitara [55]. The MARV1967 cluster (MARV80Ken, MARV12Uga, MARV67Ger and MARV08Net) and the MARV1975 cluster (MARV9800DRC, MARV14Uga and MARV75SA), apart from exported cases, were mostly concentrated in Central Africa, with inferred emergence times of approximately 1959 CE and 1910 CE, respectively. The MARV1975 cluster indicates a clear speciation event from Central Africa to South Africa. Around 1910, both regions were undergoing European colonization in the context of the New Imperialism (1810–1914) [56]. The MARV2004 cluster (MARV21Gui, MARV22Gha and MARV0405Ang), with an estimated emergence time of 1959CE (concurrent with MARV1967), showed a dispersal pattern oriented toward West Africa. Its phylogenetic placement clearly separates the Angolan strain (MARV0405Ang) from those of Ghana (MARV22Gha) and Guinea (MARV21Gui).

BOMV, which shares the same filoviral ancestor with EBOV, TAIV, and BDBV, was inferred to have speciated around 769 CE, based on its identification in a bat of the genus *Chaerophon* in Sierra Leone [24]. At that time, and geographically close to present-day Sierra Leone, the Kingdom of Ghana (500–1200 CE) was flourishing, with gold exploitation and trade representing significant activities [57,58]. On the opposite side of the split, TAIV and BDBV share a common ancestor, with divergence estimated around 1112 CE. These strains were identified in distinct regions, TAIV94CdI in Côte d’Ivoire, BDBV07Uga in Uganda, and BDBV12DRC in the Democratic Republic of the Congo. Côte d’Ivoire at that time was geographically close to the Kingdom of Ghana and the emerging Mali Empire (1235–1660 CE). In contrast, the regions corresponding to Uganda and the DRC were initially inhabited by Bantu populations (2000 BCE–500 CE), who had migrated from their homeland in present-day Nigeria [58], approximately 2513.6 km from Côte d’Ivoire [59]. By the inferred period (1112 CE), Uganda was in the early stages of power organization by Bantu clans, not yet consolidated into a kingdom, although the structure that would become the Buganda Kingdom was already forming [60]. Archeological evidence presented by Oslisly et al. (2013) indicates that a dramatic population decline occurred in Central Africa (Congo Basin) between 1000 and 1300 CE [61]. Considering that the current case fatality rate of BDBV is approximately 30% in humans [13], and that the inferred divergence coincides with this period, it is not implausible that a filovirus of the BDBV/TAIV lineage may have contributed to this historical demographic collapse.

RESV comprises two main clusters, RESV1989 (RESV89Phi and RESV89USA) and RESV1992 (RESV96USA, RESV92Ita and RESV08Phi). Excluding the export cases to Italy and the United States, the emergence of these clusters originated in the Philippines. Considering that the inferred ancestral emergence coincides with the isolation period (1989 CE), and following the contextual patterns already discussed for other filoviral lineages, the Philippines at that time faced a critical scenario of environmental degradation, primarily driven by intensive natural resource exploitation and the occurrence of major natural disasters [62].

Regarding the two SUDV clusters, SUDV1976 (SUDV76Sud, SUDV04Sud and SUDV79Sud) and SUDV2000 (SUDV00Uga, SUDV11Uga and SUDV12Uga), a clear geographic division is observed between strains from Uganda and Sudan, suggesting a central–northern distribution in Africa. The inferred divergence time is approximately 1921 CE. At that time, both Uganda and Sudan were under the British Protectorate, experiencing the expansion of cash crops such as cotton and coffee, which profoundly altered land use. This expansion brought agricultural areas into closer contact with mosaics of wooded savannas, riparian forests, and limestone caves, habitats ideally suited for large colonies of pteropodid bats such as *Rousettus aegyptiacus* [51,56,63]. This context is corroborated by the emergence of the SUDV76Sud strain, which was associated with bats in a cotton factory in Nzara [64].

Exploring the five EBOV clusters, it is possible to follow a chronology consistent with the temporal inferences obtained. The EBOV20DRC strain, isolated within the EBOV branch and with an estimated emergence date of 1937, anchors the likely ancestral lineage of the species in Central Africa. Since the Congo Basin has been exploited in multiple ways since the late 15th century, several environmental changes may be linked to this emergence, ranging from population movements to intensive resource extraction, exemplified by the development of the Central African Copperbelt around the same period [65,66]. The EBOV1976 cluster (EBOV76DRC, EBOV77DRC and EBOV17DRC) and the EBOV1994 cluster (EBOV95DRC, EBOV94Gab, EBOV96Gab, EBOV14DRC and EBOV18DRC) show divergence times consistent with their detection periods, maintaining their endemic position in Central Africa. The EBOV2001 cluster (EBOV01Gab, EBOV03RC, EBOV07DRC and EBOV08DRC) follows the same pattern. However, its ancestor gave rise around 1981 CE to the EBOV2014 lineage (EBOV14Mal, EBOV21Gui, EBOV14UK, EBOV14Ita, EBOV14USA and EBOV14Gui). Excluding non-autochthonous cases, this cluster, associated with the 2013–2016 West African outbreak [1], indicates a dispersal towards northwestern Africa, reinforcing the hypothesis of species origin in the Congo Basin. By the 1980s, corresponding to the inferred divergence of EBOV2014, much of Africa was experiencing the severe consequences of colonization and decolonization, marked by civil wars, coups d’État, genocides, and ethnic and religious conflicts [67].

Therefore, based on the temporal inferences presented, filoviral ancestors can be didactically classified into three groups:

Ancestral lineage (before 1 MYA): *Thamnovirus percae* (FIWV), *Thamnovirus kanderense* (KANV), *Thamnovirus thamnaconi* (HUJV), *Oblavirus percae* (OBLV), *Striavirus antennarii* (XILV) and *Tapjovirus bothropis* (TAPV);

Intermediate lineage (BCE to 1 MYA): *Cuevavirus lloviuense* (LLOV) and *Dianlovirus menglaense* (MLAV).

Contemporary lineage (CE): *Orthomarburgvirus marburgense* (MARV), *Orthoebolavirus sudanense* (SUDV), *Orthoebolavirus restonense* (RESV), *Orthoebolavirus bombaliense* (BOMV), *Orthoebolavirus bundibugyoense* (BDBV), *Orthoebolavirus taiense* (TAIV) and *Orthoebolavirus zairense* (EBOV).

This classification may prove useful for historical-epidemiological studies involving paleovirology and paleopathology. Although unprecedented, the temporal inferences presented here should be regarded as exploratory proposals. Future studies with more robust methodologies will be developed for a deeper exploration of these inferences.

Regarding the statistical analyses of the clusters formed in the phylogeny of filoviral proteome sequences, significant results were observed linking the groupings to the number of human cases, deaths, and case fatality. These findings suggest that phylogenetic structure may play a partial role in shaping epidemiological impact. All statistical comparisons were corrected for multiple testing using the Benjamini–Hochberg method (FDR), which reduces the likelihood of false positives and helps stabilize results in the presence of multiple comparisons [68]. Nevertheless, this approach does not completely eliminate the intrinsic effects of uneven sample sizes. In addition, the analyses were based on reference and available sequences from public databases, which are naturally biased toward lineages with major human outbreaks, particularly *Orthoebolavirus zairense* (EBOV), while bat-derived or less-studied filoviruses remain underrepresented due to the scarcity of deposited protein sequences. Although groups with a higher number of sequences, such as EBOV, benefit from greater statistical power, clusters with few representatives are limited in their ability to reveal significant associations. This methodological trade-off reinforces the exploratory nature of our analyses, in which the priority was to preserve the representativeness of all taxa with known or potential human epidemiological relevance.

In terms of the number of human cases, the clusters EBOV1994 (*p* = 0.009), EBOV2001 (*p* = 0.048), and FishFilo (*p* = 0.004) were significant. The first two are associated with a meaningful pattern of filoviral evolutionary dispersal, making these lineages particularly relevant for public health surveillance. The FishFilo cluster, on the other hand, indicates the opposite, as no cases have been recorded in humans or even in mammals.

Regarding the number of deaths, the clusters with significant associations were RESV1992 (*p* = 0.034), EBOV1994 (*p* = 0.006), EBOV2001 (*p* = 0.033) and FishFilo (*p* = 0.013). The EBOV clusters, in addition to showing statistical evidence of dispersal, also demonstrate relevant pathogenic potential. In contrast, RESV and FishFilo exhibit the opposite significance. The RESV1992 cluster, although associated with asymptomatic human infections [13], has no recorded fatalities, and this finding corroborates the low pathogenic potential of RESV in humans [69].

Regarding case fatality, the clusters RESV1992 (*p* = 0.035), MARV2004 (*p* = 0.036) and FishFilo (*p* = 0.014) showed significant associations. RESV and FishFilo fit within the patterns previously discussed, whereas MARV2004 stands out in the opposite direction, positioning this lineage as possibly the most dangerous public health threat in that variable among the currently known members of the *Filoviridae* family.

For the clusters/singletons that did not show significant associations with epidemiological data, it can be suggested that factors such as geographic context, public health response and filoviral ecology are more decisive in determining the number of cases, deaths and case fatality, thereby corroborating the previous discussion.

### 4.2. GP Phase

The evolutionary distances in this experimental phase were calculated using the Jones, Taylor and Thornton (1992) method, which is regarded as efficient for generating substitution matrices from a large number of protein sequences [70].

Based on filoviruses isolated from mammals, the GP, or glycoprotein, is a trimeric class I transmembrane protein, also functioning as a class I fusion protein composed of the GP1 and GP2 subunits linked by a disulfide bond. Its functions are associated with viral adsorption to susceptible cells, induction of membrane fusion (virus–cell), determination of cell tropism and inhibition of intrinsic immune responses [1].

Considering that only seven clusters/singletons exhibited robust phylogenetic reconstructions in this experimental phase, these difficulties can likely be attributed to the high selective pressure acting on GP [71,72].

The possible sharing of an ancestral GP between TAIV and BDBV supports previously suggested hypotheses regarding the dispersal of this lineage, considering only human hosts and the corresponding epidemiological data. This finding highlights a potential fitness advantage of BDBV over TAIV.

The ancestral sharing in GP formation between MARV and MLAV may outline a potential filoviral dispersal scenario from Asia to Africa, considering that they represent a contemporary and an intermediate lineage, respectively. However, new filoviral identifications may easily reshape this evolutionary scenario.

SUDV maintained its clear dispersal pattern between strains from Uganda and Sudan. In RESV, the isolation of the RESV08Phi strain is noteworthy, this GP reconstruction is consistent with the six asymptomatic human cases, in which the potential spillover originated from swine, whereas in the RESV89Phi strain the spillover occurred from non-human primates [13].

Regarding the FishFilo cluster, it grouped with a Bootstrap value of 98, indicating not only robustness in the evolutionary reconstruction of its GPs but also that its distance from mammalian filovirus clusters/singletons such as MARV, MLAV, TAIV, and BDBV supports its proposed temporal classification relative to the others, as an ancestral lineage (filoviruses predating 1 MYA).

Concerning the statistical findings of the GP phase, significant differences were observed in certain clusters. The FishFilo cluster showed significantly distinct values in the number of deaths, an expected outcome given the absence of clinical records in humans, which contrasts sharply with the other filoviruses. RESV also stood out, presenting a significant difference in the number of deaths for the same reason as FishFilo. In contrast, the significant difference observed in EBOV1994 should be interpreted with caution, since with a bootstrap support of only 52, its evolutionary position is uncertain enough that the statistical results may not accurately reflect reality.

### 4.3. L Phase

The Large protein (L) is part of the filoviral ribonucleoprotein complex (RNP), serving as a component of the viral inclusion body and the catalytic center of the polymerase (when associated with VP35). It contains binding regions for filoviral genomes and antigenomes and can also interact directly with VP35 and VP30. As an RNA-dependent RNA polymerase (RdRp), L belongs to an exclusive group of proteins known as Virus Hallmark Proteins, which are widely distributed across viruses and considered defining features of the viral state [73]. RdRps are distributed throughout the domain *Orthornavirae* [74]. Within the *Filoviridae* family, its functions include genome replication, messenger RNA synthesis, capping, methylation and polyadenylation [1,75].

The phylogenetic reconstruction of the L protein (Figure S2) corroborated most of the topology observed in the proteome-based filoviral reconstruction (Figure 1), with the exception of some subclusters that were split into singletons within EBOV and MARV, which nonetheless retained their positions within the discussed context. However, an inversion was observed compared to the proteome tree in the L-based reconstruction, the positions of XILV11Chi (Bootstrap 69) and TAPV18Bra (Bootstrap 100) were reversed. Nevertheless, Horie (2021) [9], also using L amino acid sequences (reference sequences only), demonstrated that TAPV and XILV form a well-supported cluster that shares ancestry with mammalian filoviruses, suggesting that the inversion observed here may not necessarily reflect the true evolutionary relationship between these lineages. This discrepancy may be explained by: (i) limitations of the phylogenetic signal of the L protein, which is relatively conserved among RNA viruses [73], making the resolution of deep branches more difficult; (ii) topological conflicts between different proteins (proteome vs. L), indicating a complex evolutionary history [76]; or (iii) long-branch artifacts associated with representing XILV and TAPV as singletons [77].

The statistical phase of L indicated no evidence that the defined clusters/singletons significantly influence the evaluated epidemiological variables.

### 4.4. NP Phase

The Nucleoprotein (NP) is also part of the RNP complex and a component of the viral inclusion body. It binds to single-stranded ribonucleic acids, as well as to VP35, VP40, VP30 and VP24. Its functions include the formation of the RNP through encapsidation of the filoviral genome, in addition to playing a central role in replication and transcription processes [1], with direct interaction with the L protein [78]. This may explain why the phylogenetic reconstruction of NP displayed a topology closely resembling that observed for L (Figure S2). However, in this reconstruction, TAPV regained the position inferred in the proteome-based analysis (sharing ancestry with the mammalian filovirus ancestor), which in turn supports the possibility of phylogenetic reconstruction error previously discussed in the L section.

SUDV was positioned in an earlier node, considering its placement in the L phase (Figure S2) and in the Proteome (Figure 1). This positioning places its nucleoproteins in speciation at the ancestor of the other *Orthoebolavirus* members. The divergence of SUDV from the same NP ancestor as the other species of the genus suggests that its protein structure is the closest to the evolutionary origin of the group in this regard. This finding is partially consistent with the results of Landeras-Bueno et al. (2019), who, through structural crystallography, revealed important differences in the NP–VP35 interaction of SUDV compared to EBOV and MARV [79].

A topographic difference from the expected evolutionary pattern was observed with BOMV, which diverged from the same ancestor as EBOV, RESV, TAIV, and BDBV, rather than EBOV, TAIV, and BDBV. This particular topography of the NP may reflect complex evolutionary processes [76]. However, its proximity to nodes associated with filoviruses non-pathogenic to humans, together with the findings of Bodmer et al. (2023), who demonstrated through in vitro experiments and reverse genetics that BOMV exhibits low pathogenicity in humans [69], suggests another possible interpretation. Based on these elements, the singleton BOMV could be considered a filoviral “fossil” (within its genus), closer to the putative zoonotic spillover event. This interpretation is also consistent with temporal inference results, which point to BOMV as possibly the oldest known species of the genus *Orthoebolavirus* (769 CE).

The statistical phase of NP indicated no evidence that the defined clusters/singletons significantly influence the evaluated epidemiological variables, corroborating the findings of the L phase.

### 4.5. VP24 Phase

The viral protein 24 (VP24) is associated with the complex RNP, functioning as a negative regulator of transcription and replication, and controlling filovirus morphogenesis and egress [1]. The genera *Oblavirus* (OBLV), *Striavirus* (XILV) and *Thamnovirus* (FIWV, KANV, HUJV) lack VP24 [7,8].

An additional function attributed to VP24 is its interaction with the nuclear transport protein karyopherins (KPNA), which shuttle STAT proteins into the nucleus to activate interferon-stimulated genes, thereby modulating host innate immunity [80,81,82]. Yu et al. (2010) showed that, unlike mammalian type I interferons, recombinant fish IFN can positively regulate its own expression, a process enhanced by Stat1 overexpression [83]. This peculiarity raises the possibility that the absence of a VP24 gene in the FishFilo cluster could be linked to such host-specific interferon dynamics.

Regarding TAPV, the presence of VP24 [9] may reflect greater similarity to mammalian immune system components. Chen et al. (2013) reported evolutionary conservation of type II interferon system features in the green anole lizard (Sauropsida: Dactyloidae: *Anolis carolinensis* Voigt, 1832) [84]. Building on this evidence, it is plausible to hypothesize that related mechanisms could also occur in other reptiles and considering the critical role of macrophages in the pathogenesis of filoviral diseases in humans [1], VP24 could represent a relevant evolutionary element in the history of the *Filoviridae* family.

The phylogenetic reconstruction of VP24 produced a topology similar to that of the NP tree (Figure S3), corroborating their functional association [85]. The topology grouped several EBOV strains within the EBOV1994 cluster, which includes lineages associated with the major 2013–2016 outbreak. However, no statistical evidence was found linking these groupings to epidemiological data, except for RESV, which showed significance for both number of deaths and case fatality. As previously discussed, this significance arises from the fact that RESV values for these variables are zero.

### 4.6. VP30 Phase

VP30 is also part of the RNP, with binding capacity to single-stranded RNA, L, and NP. Its function is associated with transcriptional regulation and RNA synthesis [1].

The EBOV cluster showed statistically significant results for both the number of cases and deaths. Within the *Filoviridae* family, *Orthoebolavirus zairense* encompasses the lineages of greatest public health concern, particularly during the major 2013–2016 outbreak [1,13]. The findings from VP30 topology and statistical analyses suggest an association with increased dispersal capacity and virulence. Supporting this interpretation, EBOV VP30 has been shown to suppress the RNA interference (RNAi) pathway [86] and regulate GP editing [87]. Collectively, these features could enhance viral fitness during biosynthesis and increase dispersal potential. To reinforce this hypothesis, VP30 in MARV has been reported to play only a minor role in transcriptional enhancement [88,89], which is consistent with the absence of significant findings in this phase.

In contrast, RESV showed significant results for cases, deaths and case fatality, mirroring the scenario observed in the Proteome phase. In this context, the significance of RESV can be interpreted as representing an opposite pattern to EBOV.

### 4.7. VP35 Phase

VP35 acts as a polymerase cofactor and is an integral component of the RNP complex [1]. Its immunomodulatory capacity, mediated by the interferon inhibitory domain (IID) [90,91,92], may help explain the statistical significance observed in the EBOV1976 cluster regarding fatalities. Notably, in EBOV1976, relatively small outbreaks showed extremely high proportions of deaths (e.g., EBOV76DRC—318 cases, 280 deaths; EBOV18DRC—3524 cases, 2320 deaths; EBOV96Gab—91 cases, 66 deaths). In contrast, the large 2013–2016 West African outbreak, despite involving 28,610 cases and 11,308 deaths (EBOV14Gui), did not yield statistical significance in the same analysis, largely because the case fatality ratio (~39.5%) was proportionally lower. This highlights how smaller but deadlier outbreaks may stand out statistically compared to large-scale epidemics with comparatively reduced lethality.

### 4.8. VP40 Phase

The matrix protein VP40 has two distinct functional domains, which assemble into dimers, linear hexamers and circular octamers, with functions associated with membrane binding, matrix filament polymerization and single-stranded RNA binding, respectively. It acts as a regulator of viral particle egress and morphogenesis and can also negatively regulate genomic replication and transcription [1].

Considering the structural rearrangements of VP40 and its impact on filoviral biosynthesis [93], the phylogenetic reconstruction points to a complex evolutionary scenario [76]. Within MARV, a division was observed (Bootstrap 100) separating the singleton MARV67Ger from the other members; however, further exploration is limited, as the cluster containing the remaining MARV isolates showed an uncertain evolutionary pattern (Bootstrap 61). A similar situation was observed in SUDV, which only in the VP40 phase lost the clear separation between Ugandan and Sudanese strains, forming a singleton (SUDV11Uga, Bootstrap 100) and a cluster of the remaining members supported by a low-confidence node (Bootstrap 55).

As the evolutionary topology resembled that of the VP30 phase, statistical results were also similar, involving the same clusters and epidemiological variables. This suggests a possible relationship between the phylogenetic reconstructions of VP30 and VP40, associating EBOV and RESV with epidemiological outcomes of cases, deaths and case fatality. At the molecular level, VP30 functions as a transcriptional initiator [1,94], whereas VP40 acts as a negative regulator of replication and transcription [1,95]. Additionally, in EBOV, both proteins independently suppress RNAi [86]. Taken together, these findings support the hypothesis that VP30–VP40 may be linked to viral dispersal (number of cases) and virulence (number of deaths) in humans for *Orthoebolavirus zairense*, highlighting the relevance of this relationship to public health. Conversely, RESV, as observed in the VP30 phase, serves as evidence in the opposite direction, reinforcing through both historical and statistical perspectives the possible particularity of the VP30–VP40, EBOV–human interaction. Nevertheless, these correlations should be regarded as exploratory signals rather than definitive associations, requiring future testing under more robust phylogenetic–epidemiological models.

Beyond their historical–evolutionary significance, the results may also serve as a base for practical applications. The proposed classification of clades and their temporal inferences can support zoonotic spillover surveillance, by identifying lineages with a stronger history of human involvement. Furthermore, this framework may assist in clade prioritization for diagnostics, guiding molecular detection tools toward epidemiologically relevant groups. Finally, the exploratory temporal trends outlined here could contribute to outbreak prediction, serving as a starting point for models of viral emergence and epidemic risk. Together, these applications reinforce the translational value of the phylogenetic approach presented in this study.

## 5. Conclusions

Temporal inferences allowed the organization of filoviral ancestors into three major groups: an ancestral lineage (>1 MYA, represented mainly by fish- and reptile-associated filoviruses), an intermediate lineage (BCE–1MYA, mammalian hosts such as bats) and a contemporary lineage (CE, encompassing ebolaviruses and marburgviruses). This proposal provides a didactic framework that may serve as a basis for paleovirology and paleopathology studies, linking viral evolution to both environmental and historical contexts.

The VP30 and VP40 phases revealed a consistent association between phylogenetic topology and epidemiological variables, particularly in *Orthoebolavirus zairense*. The complementary roles of these proteins, VP30 as a transcriptional initiator and VP40 as a negative regulator of replication and viral morphogenesis, may be linked to increased dispersal (cases) and virulence (fatalities). This functional interplay should be regarded as an exploratory but evidence-supported signal, pointing to a promising possibility for future investigations in molecular virology and public health.

*Orthoebolavirus restonense* acting as a natural counterpoint for comparison with other potential human pathogenic filoviruses.

Therefore, the integrated analysis revealed that topological robustness does not always correspond to epidemiological stability. In some phases, consistent topologies (such as Proteome and L) contrasted with proteins subjected to stronger selective pressures (GP, VP30, VP40). Moreover, temporal inferences connect filoviruses not only to the recent history of human epidemics but also, based on available evidence, to deeper ecological events, from the formation of the Pebas system in the Amazon to European colonization in Africa. This perspective highlights that filoviral evolution is intrinsically tied to both the molecular biology of filoviruses and the ecology and history of their hosts.

## Figures and Tables

**Table 1:** Temporal inference of the family Filoviridae.

Species/Abbreviation	Approximate Time
*Orthoebolavirus zairense*/EBOV	1937–1981 CE
*Orthoebolavirus taiense*/TAIV	1112 CE
*Orthoebolavirus bundibugyoense*/BDBV	1112 CE
*Orthoebolavirus bombaliense*/BOMV	769 CE
*Orthoebolavirus restonense*/RESV	1989 CE
*Orthoebolavirus sudanense*/SUDV	1921 CE
*Orthomarburgvirus marburgense*/MARV	1773–1959 CE
*Cuevavirus lloviuense*/LLOV	3433 BCE
*Dianlovirus menglaense*/MLAV	1569 BCE
*Tapjovirus bothropis*/TAPV	10 MYA
*Striavirus antennarii*/XILV	11 MYA
*Oblavirus percae*/OBLV	32 MYA
*Thamnovirus thamnaconi*/HUJV	32 MYA
*Thamnovirus kanderense*/KANV	32 MYA
*Thamnovirus percae*/FIWV	32 MYA

CE: Common Era; BCE: Before the Common Era; MYA: Millions of Years Ago.

## Data Availability

The original contributions presented in this study are included in the article/Supplementary Materials. Further inquiries can be directed to the corresponding author.

## Supplementary Materials

The following supporting information can be downloaded at: https://www.mdpi.com/article/10.3390/microorganisms13102388/s1, Supplementary Files S1–S27 are related to phylogenetic reconstruction, presenting the parameters applied in the experimental phases. The File S28: Supplementary Figures’ Captions contains the corresponding legends for Supplementary Figures S1–S7. Table S1 (Experimental Phases) provides the sequence identifiers, their associated epidemiological data and the bootstrap values obtained from the phylogenetic reconstruction. Figures S1–S7 display the filoviral phylogenetic trees corresponding to the GP, L, NP, VP24, VP30, VP35 and VP40 proteins.
