# Supplementary material for: Exploratory Temporal and Evolutionary Insights into the Filoviridae Family Through Multiprotein Phylogeny
Source: Microorganisms. 2025 Oct 17;13(10):2388. doi: 10.3390/microorganisms13102388 (PMC12566026; doi:10.3390/microorganisms13102388)
Supplement: Supplementary file 1 [file microorganisms-13-02388-s001.zip › File S28 Supplementary Figures’ captions.pdf]

File S28: Supplementary Figures' captions

Figure S1. Filoviral Phylogenetic Tree (GP). *Orthoebolavirus zairense* (EBOV) cluster in green, *Orthoebolavirus restonense* (RESV) cluster in yellow, Orthoebolavirus sudanense (SUDV) cluster in orange, *Orthomarburgvirus marburgense* (MARV) cluster in purple, FishFilo cluster in blue. Taxon IDs: MARV67Ger—MARV Germany 1967; MARV75SA—MARV South Africa 1975; SUDV76Sud—SUDV Sudan 1976; EBOV76DRC—EBOV Democratic Republic of the Congo 1976; EBOV77DRC—EBOV Democratic Republic of the Congo 1977; SUDV79Sud—SUDV Sudan 1979; MARV80Ken—MARV Kenya 1980; MARV87Ken—MARV Kenya 1987; RESV89Phi—RESV Philippines 1989; RESV89USA—RESV United States of America 1989; RESV92Ita—RESV Italy 1992; TAIV94CdI—*Orthoebolavirus taiense* (TAIV) Côte d'Ivoire 1994; EBOV94Gab—EBOV Gabon 1994; EBOV95DRC—EBOV Democratic Republic of the Congo 1995; RESV96USA—RESV United States of America 1996; EBOV96Gab—EBOV Gabon 1996; MARV9800DRC—MARV Democratic Republic of the Congo 1998–2000; SUDV00Uga—SUDV Uganda 2000; EBOV01Gab—EBOV Gabon 2001; EBOV03RC—EBOV Republic of the Congo 2003; MARV0405Ang—MARV Angola 2004–2005; SUDV04Sud—SUDV Sudan 2004; MARV07Uga—MARV Uganda 2007; BDBV07Uga—*Orthoebolavirus bundibugyoense* (BDBV) Uganda 2007; EBOV07DRC—EBOV Democratic Republic of the Congo 2007; MARV08Net—MARV Netherlands 2008; EBOV08DRC—EBOV Democratic Republic of the Congo 2008; RESV08Phi—RESV Philippines 2008; SUDV11Uga—SUDV Uganda 2011; MARV12Uga—MARV Uganda 2012; SUDV12Uga—SUDV Uganda 2012; BDBV12DRC—BDBV Democratic Republic of the Congo 2012; MARV14Uga—MARV Uganda 2014; EBOV14DRC—EBOV Democratic Republic of the Congo 2014; EBOV14Gui—EBOV Guinea 2014; EBOV14Ita—EBOV Italy 2014; EBOV14Mal—EBOV Mali 2014; EBOV14UK—EBOV United Kingdom 2014; EBOV14USA—EBOV United States of America 2014; EBOV17DRC—EBOV Democratic Republic of the Congo 2017; EBOV18DRC—EBOV Democratic Republic of the Congo 2018; EBOV20DRC—EBOV Democratic Republic of the Congo 2020; MARV21Gui—MARV Guinea 2021; EBOV21Gui—EBOV Guinea 2021; MARV22Gha—MARV Ghana 2022; LLOV03Spa—*Cuevavirus lloviuense* (LLOV) Spain 2003; MLAV15Chi—*Dianlovirus menglaense* (MLAV) China 2015; BOMV16SL—*Orthoebolavirus bombaliense* (BOMV) Sierra Leone 2016; TAPV18Bra—*Tapjovirus bothropis* (TAPV) Brazil 2018; OBLV17Swi—*Oblavirus percae* (OBLV) Switzerland 2017; KANV16Swi—*Thamnovirus kanderense* (KANV) Switzerland 2016; XILV11Chi—*Striavirus antennarii* (XILV) China 2011; FIWV17Swi—*Thamnovirus percae* (FIWV) Switzerland 2017; HUJV11Chi—*Thamnovirus thamnaconi* (HUJV) China 2011; RABV—*Lyssavirus rabies*.

Figure S2. Filoviral Phylogenetic Tree (L). *Orthoebolavirus zairense* (EBOV) cluster in green, *Orthoebolavirus restonense* (RESV) cluster in yellow, *Orthoebolavirus sudanense* (SUDV) cluster in orange, *Orthomarburgvirus marburgense* (MARV) cluster in purple, FishFilo cluster in blue. Taxon IDs: MARV67Ger—MARV Germany 1967;

MARV75SA—MARV South Africa 1975; SUDV76Sud—SUDV Sudan 1976; EBOV76DRC—EBOV Democratic Republic of the Congo 1976; EBOV77DRC—EBOV Democratic Republic of the Congo 1977; SUDV79Sud—SUDV Sudan 1979; MARV80Ken—MARV Kenya 1980; MARV87Ken—MARV Kenya 1987; RESV89Phi—RESV Philippines 1989; RESV89USA—RESV United States of America 1989; RESV92Ita—RESV Italy 1992; TAIV94CdI—*Orthoebolavirus taiense* (TAIV) Côte d'Ivoire 1994; EBOV94Gab—EBOV Gabon 1994; EBOV95DRC—EBOV Democratic Republic of the Congo 1995; RESV96USA—RESV United States of America 1996; EBOV96Gab—EBOV Gabon 1996; MARV9800DRC—MARV Democratic Republic of the Congo 1998–2000; SUDV00Uga—SUDV Uganda 2000; EBOV01Gab—EBOV Gabon 2001; EBOV03RC—EBOV Republic of the Congo 2003; MARV0405Ang—MARV Angola 2004–2005; SUDV04Sud—SUDV Sudan 2004; MARV07Uga—MARV Uganda 2007; BDBV07Uga—*Orthoebolavirus bundibugyoense* (BDBV) Uganda 2007; EBOV07DRC—EBOV Democratic Republic of the Congo 2007; MARV08Net—MARV Netherlands 2008; EBOV08DRC—EBOV Democratic Republic of the Congo 2008; RESV08Phi—RESV Philippines 2008; SUDV11Uga—SUDV Uganda 2011; MARV12Uga—MARV Uganda 2012; SUDV12Uga—SUDV Uganda 2012; BDBV12DRC—BDBV Democratic Republic of the Congo 2012; MARV14Uga—MARV Uganda 2014; EBOV14DRC—EBOV Democratic Republic of the Congo 2014; EBOV14Gui—EBOV Guinea 2014; EBOV14Ita—EBOV Italy 2014; EBOV14Mal—EBOV Mali 2014; EBOV14UK—EBOV United Kingdom 2014; EBOV14USA—EBOV United States of America 2014; EBOV17DRC—EBOV Democratic Republic of the Congo 2017; EBOV18DRC—EBOV Democratic Republic of the Congo 2018; EBOV20DRC—EBOV Democratic Republic of the Congo 2020; MARV21Gui—MARV Guinea 2021; EBOV21Gui—EBOV Guinea 2021; MARV22Gha—MARV Ghana 2022; LLOV03Spa—*Cuevavirus lloviuense* (LLOV) Spain 2003; MLAV15Chi—*Dianlovirus menglaense* (MLAV) China 2015; BOMV16SL—*Orthoebolavirus bombaliense* (BOMV) Sierra Leone 2016; TAPV18Bra—*Tapjovirus bothropis* (TAPV) Brazil 2018; OBLV17Swi—*Oblavirus percae* (OBLV) Switzerland 2017; KANV16Swi—*Thamnovirus kanderense* (KANV) Switzerland 2016; XILV11Chi—*Striavirus antennarii* (XILV) China 2011; FIWV17Swi—*Thamnovirus percae* (FIWV) Switzerland 2017; HUJV11Chi—*Thamnovirus thamnaconi* (HUJV) China 2011; RABV—*Lyssavirus rabies*.

Figure S3. Filoviral Phylogenetic Tree (NP). *Orthoebolavirus zairense* (EBOV) cluster in green, *Orthoebolavirus restonense* (RESV) cluster in yellow, *Orthoebolavirus sudanense* (SUDV) cluster in orange, *Orthomarburgvirus marburgense* (MARV) cluster in purple, FishFilo cluster in blue. Taxon IDs: MARV67Ger—MARV Germany 1967; MARV75SA—MARV South Africa 1975; SUDV76Sud—SUDV Sudan 1976; EBOV76DRC—EBOV Democratic Republic of the Congo 1976; EBOV77DRC—EBOV Democratic Republic of the Congo 1977; SUDV79Sud—SUDV Sudan 1979; MARV80Ken—MARV Kenya 1980; MARV87Ken—MARV Kenya 1987; RESV89Phi—RESV Philippines 1989; RESV89USA—RESV United States of America 1989; RESV92Ita—RESV Italy 1992; TAIV94CdI—*Orthoebolavirus taiense* (TAIV) Côte d'Ivoire 1994; EBOV94Gab—EBOV Gabon 1994; EBOV95DRC—EBOV Democratic Republic of the Congo 1995; RESV96USA—RESV United States of

America 1996; EBOV96Gab—EBOV Gabon 1996; MARV9800DRC—MARV Democratic Republic of the Congo 1998–2000; SUDV00Uga—SUDV Uganda 2000; EBOV01Gab—EBOV Gabon 2001; EBOV03RC—EBOV Republic of the Congo 2003; MARV0405Ang—MARV Angola 2004–2005; SUDV04Sud—SUDV Sudan 2004; MARV07Uga—MARV Uganda 2007; BDBV07Uga—*Orthoebolavirus bundibugyoense* (BDBV) Uganda 2007; EBOV07DRC—EBOV Democratic Republic of the Congo 2007; MARV08Net—MARV Netherlands 2008; EBOV08DRC—EBOV Democratic Republic of the Congo 2008; RESV08Phi—RESV Philippines 2008; SUDV11Uga—SUDV Uganda 2011; MARV12Uga—MARV Uganda 2012; SUDV12Uga—SUDV Uganda 2012; BDBV12DRC—BDBV Democratic Republic of the Congo 2012; MARV14Uga—MARV Uganda 2014; EBOV14DRC—EBOV Democratic Republic of the Congo 2014; EBOV14Gui—EBOV Guinea 2014; EBOV14Ita—EBOV Italy 2014; EBOV14Mal—EBOV Mali 2014; EBOV14UK—EBOV United Kingdom 2014; EBOV14USA—EBOV United States of America 2014; EBOV17DRC—EBOV Democratic Republic of the Congo 2017; EBOV18DRC—EBOV Democratic Republic of the Congo 2018; EBOV20DRC—EBOV Democratic Republic of the Congo 2020; MARV21Gui—MARV Guinea 2021; EBOV21Gui—EBOV Guinea 2021; MARV22Gha—MARV Ghana 2022; LLOV03Spa—*Cuevavirus lloviuense* (LLOV) Spain 2003; MLAV15Chi—*Dianlovirus menglaense* (MLAV) China 2015; BOMV16SL—*Orthoebolavirus bombaliense* (BOMV) Sierra Leone 2016; TAPV18Bra—*Tapjovirus bothropis* (TAPV) Brazil 2018; OBLV17Swi—*Oblavirus percae* (OBLV) Switzerland 2017; KANV16Swi—*Thamnovirus kanderense* (KANV) Switzerland 2016; XILV11Chi—*Striavirus antennarii* (XILV) China 2011; FIWV17Swi—*Thamnovirus percae* (FIWV) Switzerland 2017; HUJV11Chi—*Thamnovirus thamnaconi* (HUJV) China 2011; RABV—*Lyssavirus rabies*.

Figure S4. Filoviral Phylogenetic Tree (VP24). *Orthoebolavirus zairense* (EBOV) cluster in green, *Orthoebolavirus restonense* (RESV) cluster in yellow, *Orthoebolavirus sudanense* (SUDV) cluster in orange, *Orthomarburgvirus marburgense* (MARV) cluster in purple. Taxon IDs: MARV67Ger—MARV Germany 1967; MARV75SA—MARV South Africa 1975; SUDV76Sud—SUDV Sudan 1976; EBOV76DRC—EBOV Democratic Republic of the Congo 1976; EBOV77DRC—EBOV Democratic Republic of the Congo 1977; SUDV79Sud—SUDV Sudan 1979; MARV80Ken—MARV Kenya 1980; MARV87Ken—MARV Kenya 1987; RESV89Phi—RESV Philippines 1989; RESV89USA—RESV United States of America 1989; RESV92Ita—RESV Italy 1992; TAIV94CdI—*Orthoebolavirus taiense* (TAIV) Côte d'Ivoire 1994; EBOV94Gab—EBOV Gabon 1994; EBOV95DRC—EBOV Democratic Republic of the Congo 1995; RESV96USA—RESV United States of America 1996; EBOV96Gab—EBOV Gabon 1996; MARV9800DRC—MARV Democratic Republic of the Congo 1998–2000; SUDV00Uga—SUDV Uganda 2000; EBOV01Gab—EBOV Gabon 2001; EBOV03RC—EBOV Republic of the Congo 2003; MARV0405Ang—MARV Angola 2004–2005; SUDV04Sud—SUDV Sudan 2004; MARV07Uga—MARV Uganda 2007; BDBV07Uga—*Orthoebolavirus bundibugyoense* (BDBV) Uganda 2007; EBOV07DRC—EBOV Democratic Republic of the Congo 2007; MARV08Net—MARV Netherlands 2008; EBOV08DRC—EBOV Democratic Republic of the Congo 2008; RESV08Phi—RESV Philippines 2008; SUDV11Uga—SUDV Uganda 2011;

MARV12Uga—MARV Uganda 2012; SUDV12Uga—SUDV Uganda 2012; BDBV12DRC—BDBV Democratic Republic of the Congo 2012; MARV14Uga—MARV Uganda 2014; EBOV14DRC—EBOV Democratic Republic of the Congo 2014; EBOV14Gui—EBOV Guinea 2014; EBOV14Ita—EBOV Italy 2014; EBOV14Mal—EBOV Mali 2014; EBOV14UK—EBOV United Kingdom 2014; EBOV14USA—EBOV United States of America 2014; EBOV17DRC—EBOV Democratic Republic of the Congo 2017; EBOV18DRC—EBOV Democratic Republic of the Congo 2018; EBOV20DRC—EBOV Democratic Republic of the Congo 2020; MARV21Gui—MARV Guinea 2021; EBOV21Gui—EBOV Guinea 2021; MARV22Gha—MARV Ghana 2022; LLOV03Spa—*Cuevavirus lloviuense* (LLOV) Spain 2003; MLAV15Chi—*Dianlovirus menglaense* (MLAV) China 2015; BOMV16SL—*Orthoebolavirus bombaliense* (BOMV) Sierra Leone 2016; TAPV18Bra—*Tapjovirus bothropis* (TAPV) Brazil 2018; RABV—*Lyssavirus rabies*.

Figure S5. Filoviral Phylogenetic Tree (VP30). *Orthoebolavirus zairense* (EBOV) cluster in green, *Orthoebolavirus restonense* (RESV) cluster in yellow, *Orthoebolavirus sudanense* (SUDV) cluster in orange, *Orthomarburgvirus marburgense* (MARV) cluster in purple. Taxon IDs: MARV67Ger—MARV Germany 1967; MARV75SA—MARV South Africa 1975; SUDV76Sud—SUDV Sudan 1976; EBOV76DRC—EBOV Democratic Republic of the Congo 1976; EBOV77DRC—EBOV Democratic Republic of the Congo 1977; SUDV79Sud—SUDV Sudan 1979; MARV80Ken—MARV Kenya 1980; MARV87Ken—MARV Kenya 1987; RESV89Phi—RESV Philippines 1989; RESV89USA—RESV United States of America 1989; RESV92Ita—RESV Italy 1992; TAIV94CdI—*Orthoebolavirus taiense* (TAIV) Côte d'Ivoire 1994; EBOV94Gab—EBOV Gabon 1994; EBOV95DRC—EBOV Democratic Republic of the Congo 1995; RESV96USA—RESV United States of America 1996; EBOV96Gab—EBOV Gabon 1996; MARV9800DRC—MARV Democratic Republic of the Congo 1998–2000; SUDV00Uga—SUDV Uganda 2000; EBOV01Gab—EBOV Gabon 2001; EBOV03RC—EBOV Republic of the Congo 2003; MARV0405Ang—MARV Angola 2004–2005; SUDV04Sud—SUDV Sudan 2004; MARV07Uga—MARV Uganda 2007; BDBV07Uga—*Orthoebolavirus bundibugyoense* (BDBV) Uganda 2007; EBOV07DRC—EBOV Democratic Republic of the Congo 2007; MARV08Net—MARV Netherlands 2008; EBOV08DRC—EBOV Democratic Republic of the Congo 2008; RESV08Phi—RESV Philippines 2008; SUDV11Uga—SUDV Uganda 2011; MARV12Uga—MARV Uganda 2012; SUDV12Uga—SUDV Uganda 2012; BDBV12DRC—BDBV Democratic Republic of the Congo 2012; MARV14Uga—MARV Uganda 2014; EBOV14DRC—EBOV Democratic Republic of the Congo 2014; EBOV14Gui—EBOV Guinea 2014; EBOV14Ita—EBOV Italy 2014; EBOV14Mal—EBOV Mali 2014; EBOV14UK—EBOV United Kingdom 2014; EBOV14USA—EBOV United States of America 2014; EBOV17DRC—EBOV Democratic Republic of the Congo 2017; EBOV18DRC—EBOV Democratic Republic of the Congo 2018; EBOV20DRC—EBOV Democratic Republic of the Congo 2020; MARV21Gui—MARV Guinea 2021; EBOV21Gui—EBOV Guinea 2021; MARV22Gha—MARV Ghana 2022; LLOV03Spa—*Cuevavirus lloviuense* (LLOV) Spain 2003; MLAV15Chi—*Dianlovirus menglaense* (MLAV) China 2015; BOMV16SL—*Orthoebolavirus bombaliense* (BOMV) Sierra Leone 2016; TAPV18Bra—*Tapjovirus bothropis* (TAPV)

Brazil 2018; XILV11Chi—*Striavirus antennarii* (XILV) China 2011; RABV—Lyssavirus rabies.

Figure S6. Filoviral Phylogenetic Tree (VP35). *Orthoebolavirus zairense* (EBOV) cluster in green, *Orthoebolavirus restonense* (RESV) cluster in yellow, *Orthoebolavirus sudanense* (SUDV) cluster in orange, *Orthomarburgvirus marburgense* (MARV) cluster in purple. Taxon IDs: MARV67Ger—MARV Germany 1967; MARV75SA—MARV South Africa 1975; SUDV76Sud—SUDV Sudan 1976; EBOV76DRC—EBOV Democratic Republic of the Congo 1976; EBOV77DRC—EBOV Democratic Republic of the Congo 1977; SUDV79Sud—SUDV Sudan 1979; MARV80Ken—MARV Kenya 1980; MARV87Ken—MARV Kenya 1987; RESV89Phi—RESV Philippines 1989; RESV89USA—RESV United States of America 1989; RESV92Ita—RESV Italy 1992; TAIV94CdI—*Orthoebolavirus taiense* (TAIV) Côte d'Ivoire 1994; EBOV94Gab—EBOV Gabon 1994; EBOV95DRC—EBOV Democratic Republic of the Congo 1995; RESV96USA—RESV United States of America 1996; EBOV96Gab—EBOV Gabon 1996; MARV9800DRC—MARV Democratic Republic of the Congo 1998–2000; SUDV00Uga—SUDV Uganda 2000; EBOV01Gab—EBOV Gabon 2001; EBOV03RC—EBOV Republic of the Congo 2003; MARV0405Ang—MARV Angola 2004–2005; SUDV04Sud—SUDV Sudan 2004; MARV07Uga—MARV Uganda 2007; BDBV07Uga—*Orthoebolavirus bundibugyoense* (BDBV) Uganda 2007; EBOV07DRC—EBOV Democratic Republic of the Congo 2007; MARV08Net—MARV Netherlands 2008; EBOV08DRC—EBOV Democratic Republic of the Congo 2008; RESV08Phi—RESV Philippines 2008; SUDV11Uga—SUDV Uganda 2011; MARV12Uga—MARV Uganda 2012; SUDV12Uga—SUDV Uganda 2012; BDBV12DRC—BDBV Democratic Republic of the Congo 2012; MARV14Uga—MARV Uganda 2014; EBOV14DRC—EBOV Democratic Republic of the Congo 2014; EBOV14Gui—EBOV Guinea 2014; EBOV14Ita—EBOV Italy 2014; EBOV14Mal—EBOV Mali 2014; EBOV14UK—EBOV United Kingdom 2014; EBOV14USA—EBOV United States of America 2014; EBOV17DRC—EBOV Democratic Republic of the Congo 2017; EBOV18DRC—EBOV Democratic Republic of the Congo 2018; EBOV20DRC—EBOV Democratic Republic of the Congo 2020; MARV21Gui—MARV Guinea 2021; EBOV21Gui—EBOV Guinea 2021; MARV22Gha—MARV Ghana 2022; LLOV03Spa—*Cuevavirus lloviuense* (LLOV) Spain 2003; MLAV15Chi—*Dianlovirus menglaense* (MLAV) China 2015; BOMV16SL—*Orthoebolavirus bombaliense* (BOMV) Sierra Leone 2016; TAPV18Bra—*Tapjovirus bothropis* (TAPV) Brazil 2018; RABV—*Lyssavirus rabies*.

Figure S7. Filoviral Phylogenetic Tree (VP40). *Orthoebolavirus zairense* (EBOV) cluster in green, *Orthoebolavirus restonense* (RESV) cluster in yellow, *Orthoebolavirus sudanense* (SUDV) cluster in orange, *Orthomarburgvirus marburgense* (MARV) cluster in purple. Taxon IDs: MARV67Ger—MARV Germany 1967; MARV75SA—MARV South Africa 1975; SUDV76Sud—SUDV Sudan 1976; EBOV76DRC—EBOV Democratic Republic of the Congo 1976; EBOV77DRC—EBOV Democratic Republic of the Congo 1977; SUDV79Sud—SUDV Sudan 1979; MARV80Ken—MARV Kenya

1980; MARV87Ken—MARV Kenya 1987; RESV89Phi—RESV Philippines 1989; RESV89USA—RESV United States of America 1989; RESV92Ita—RESV Italy 1992; TAIV94CdI—*Orthoebolavirus taiense* (TAIV) Côte d'Ivoire 1994; EBOV94Gab—EBOV Gabon 1994; EBOV95DRC—EBOV Democratic Republic of the Congo 1995; RESV96USA—RESV United States of America 1996; EBOV96Gab—EBOV Gabon 1996; MARV9800DRC—MARV Democratic Republic of the Congo 1998–2000; SUDV00Uga—SUDV Uganda 2000; EBOV01Gab—EBOV Gabon 2001; EBOV03RC—EBOV Republic of the Congo 2003; MARV0405Ang—MARV Angola 2004–2005; SUDV04Sud—SUDV Sudan 2004; MARV07Uga—MARV Uganda 2007; BDBV07Uga—*Orthoebolavirus bundibugyoense* (BDBV) Uganda 2007; EBOV07DRC—EBOV Democratic Republic of the Congo 2007; MARV08Net—MARV Netherlands 2008; EBOV08DRC—EBOV Democratic Republic of the Congo 2008; RESV08Phi—RESV Philippines 2008; SUDV11Uga—SUDV Uganda 2011; MARV12Uga—MARV Uganda 2012; SUDV12Uga—SUDV Uganda 2012; BDBV12DRC—BDBV Democratic Republic of the Congo 2012; MARV14Uga—MARV Uganda 2014; EBOV14DRC—EBOV Democratic Republic of the Congo 2014; EBOV14Gui—EBOV Guinea 2014; EBOV14Ita—EBOV Italy 2014; EBOV14Mal—EBOV Mali 2014; EBOV14UK—EBOV United Kingdom 2014; EBOV14USA—EBOV United States of America 2014; EBOV17DRC—EBOV Democratic Republic of the Congo 2017; EBOV18DRC—EBOV Democratic Republic of the Congo 2018; EBOV20DRC—EBOV Democratic Republic of the Congo 2020; MARV21Gui—MARV Guinea 2021; EBOV21Gui—EBOV Guinea 2021; MARV22Gha—MARV Ghana 2022; LLOV03Spa—*Cuevavirus lloviuense* (LLOV) Spain 2003; MLAV15Chi—*Dianlovirus menglaense* (MLAV) China 2015; BOMV16SL—*Orthoebolavirus bombaliense* (BOMV) Sierra Leone 2016; TAPV18Bra—*Tapjovirus bothropis* (TAPV) Brazil 2018; XILV11Chi—*Striavirus antennarii* (XILV) China 2011; RABV—*Lyssavirus rabies*.
