# Supplementary material for: Exploratory Temporal and Evolutionary Insights into the Filoviridae Family Through Multiprotein Phylogeny
Source: Microorganisms. 2025 Oct 17;13(10):2388. doi: 10.3390/microorganisms13102388 (PMC12566026; doi:10.3390/microorganisms13102388)
Supplement: Supplementary file 1 [file microorganisms-13-02388-s001.zip › File S1 Phylogenetic Reconstruction.pdf]

File S1: Phylogenetic Reconstruction

Proteome phase (Table S1: Experimental phases, Proteome spreadsheet):

A – Amino acid sequences were manually curated (text editing and removal of "*" characters); the sequence NC_001542.1 (*Lyssavirus rabies*) was included as outgroup;

B – Alignment was performed using the MUSCLE algorithm (Multiple Sequence Comparison by Log-Expectation) under default settings;

C – Model testing was conducted by Maximum Likelihood (ML) with the following configuration: Automatic (Neighbor-joining tree), Approach Full (Slow), Gaps/Missing Data Complete deletion, Branch Swap Filter Strong, Number of Threads 4 (File S2: Model Testing Proteome);

D – Phylogenetic reconstruction was performed with the following parameters: Adaptive Bootstrap (threshold 0.05), Maximum Likelihood method, LG+F model, Rates among Sites: Gamma Distributed (G) with 5 categories, Gaps/Missing Data: Complete deletion, Heuristic Method: Nearest-Neighbor-Interchange (NNJ), Branch Swap Filter Strong, Number of Threads 4 (File S3: Proteome Tree MTSX; File S4: Proteome Tree NWK);

E – Clusters were defined based on tree topology (Cluster/Singleton column);

F – Bootstrap values associated with the ancestral node of each cluster were recorded (Bootstrap II column);

G – Molecular clock testing was conducted with the following settings: Maximum Likelihood, Tree to Use: tree obtained in step D, LG+F model, Rates among Sites: Gamma Distributed (G) with 5 categories, Gaps/Missing Data: Complete deletion, Number of Threads 4. Output was exported as text (File S5: Test Molecular Clock (ML));

H – Temporal calibrations were set as follows: filovirus ancestral node – uniform distribution 28–400 MYA (Taylor; Barnhart, 2024); divergence of *Orthoebolavirus* and *Orthomarburgvirus* – uniform distribution 7100–7900 years (Suzuki; Gojobori, 1997); minimum times for clusters, based on the present (2025) and the earliest identified/isolated virus: EBOV2014 (11 yrs), EBOV2001 (24 yrs), EBOV1976 (49 yrs), EBOV1994 (31 yrs), TAIV/BDBV (31 yrs), RESV1989 (36 yrs), RESV1992 (33 yrs), SUDV1976 (49 yrs), SUDV2000 (25 yrs), MARV1987 (38 yrs), MARV1975 (50 yrs), MARV2004 (21 yrs), MARV1967 (58 yrs);

I – Temporal inference was performed using RelTime with the following parameters: Statistical Method: Maximum Likelihood, Tree to Use: tree from step D, LG+F model, Rates among Sites: Gamma Distributed (G) with 5 categories, Gaps/Missing Data: Complete deletion, Number of Threads 4 (File S6: Proteome Temporal Tree MTSX).

GP phase (Table S1: Experimental phases, GP spreadsheet):

A – Amino acid sequences were manually curated (text editing and removal of "*" characters); the sequence NC_001542.1 (*Lyssavirus rabies*) was included as outgroup;

B – Alignment was performed using the MUSCLE algorithm (Multiple Sequence Comparison by Log-Expectation) under default settings;

C – Model testing was conducted by Maximum Likelihood (ML) with the following configuration: Automatic (Neighbor-joining tree), Approach Full (Slow), Gaps/Missing Data Complete deletion, Branch Swap Filter Strong, Number of Threads 4 (File S7: Model Testing GP);

D – Phylogenetic reconstruction was performed with the following parameters: Bootstrap replicates 1000, Model/Method Neighbor-joining and Jones-Taylor-Thornton (JTT) model, Rates among Sites Gamma Distributed (G) with 5 categories, Gaps/Missing Data Complete deletion, ML Heuristic Method: Nearest-Neighbor-Interchange (NNJ), Branch Swap Filter Strong, Number of Threads 4 (File S8: GP Tree MTSX; File S9: GP Tree NWK);

E – Clusters/singletons were defined based on tree topology (Cluster/Singleton column);

F – Bootstrap values corresponding to the ancestor of each cluster/singleton were recorded (Bootstrap II column).

L phase (Table S1: Experimental phases, L spreadsheet):

A – Amino acid sequences were manually curated (text editing). The sequence NC_001542.1 (*Lyssavirus rabies*) was included as outgroup;

B – Alignment was performed using the MUSCLE algorithm (Multiple Sequence Comparison by Log-Expectation) under default settings;

C – Model testing was conducted by Maximum Likelihood (ML) with the following configuration: Automatic (Neighbor-joining tree), Approach Full (Slow), Gaps/Missing Data Complete deletion, Branch Swap Filter Strong, Number of Threads 4 (File S10: Model Testing L);

D – Phylogenetic reconstruction was performed with the following parameters: Adaptive Bootstrap (threshold 0.05), Maximum Likelihood method, LG+F model, Rates among Sites: Gamma Distributed (G) with 5 categories, Gaps/Missing Data: Complete deletion, Heuristic Method: Nearest-Neighbor-Interchange (NNJ), Branch Swap Filter Strong, Number of Threads 4 (File S11: L Tree MTSX; File S12: L Tree NWK);

E – Clusters/singletons were defined based on tree topology (Cluster/Singleton column);

F – Bootstrap values corresponding to the ancestor of each cluster/singleton were recorded (Bootstrap II column).

NP phase (Table S1: Experimental phases, NP spreadsheet):

A – Amino acid sequences were manually curated (text editing). The sequence NC_001542.1 (*Lyssavirus rabies*) was included as outgroup;

B – Alignment was performed using the MUSCLE algorithm (Multiple Sequence Comparison by Log-Expectation) under default settings;

C – Model testing was conducted by Maximum Likelihood (ML) with the following configuration: Automatic (Neighbor-joining tree), Approach Full (Slow), Gaps/Missing Data Complete deletion, Branch Swap Filter Strong, Number of Threads 4 (File S13: Model Testing NP);

D – Phylogenetic reconstruction was performed with the following parameters: Adaptive Bootstrap (threshold 0.05), Maximum Likelihood method, LG model, Rates among Sites: Gamma Distributed (G) with 5 categories, Gaps/Missing Data: Complete deletion, Heuristic Method: Nearest-Neighbor-Interchange (NNJ), Branch Swap Filter Strong, Number of Threads 4 (File S14: NP Tree MTSX; File S15: NP Tree NWK);

E – Clusters/singletons were defined based on tree topology (Cluster/Singleton column);

F – Bootstrap values corresponding to the ancestor of each cluster/singleton were recorded (Bootstrap II column).

VP24 phase (Table S1: Experimental phases, VP24 spreadsheet):

A – Amino acid sequences were manually curated (text editing). The sequence of the M (Matrix) protein NC_001542.1 (*Lyssavirus rabies*) was included as outgroup, and the following species were excluded: *Striavirus antennarii* (XILV), *Thamnovirus kanderense* (KANV), *Thamnovirus percae* (FIWV), *Thamnovirus thamnaconi* (HUJV), and *Oblavirus percae* (OBLV);

B – Alignment was performed using the MUSCLE algorithm (Multiple Sequence Comparison by Log-Expectation) under default settings;

C – Model testing was conducted by Maximum Likelihood (ML) with the following configuration: Automatic (Neighbor-joining tree), Approach Full (Slow), Gaps/Missing Data Complete deletion, Branch Swap Filter Strong, Number of Threads 4 (File S16: Model Testing VP24);

D – Phylogenetic reconstruction was performed with the following parameters: Adaptive Bootstrap (threshold 0.05), Maximum Likelihood method, LG model, Rates among Sites: Gamma Distributed (G) with 5 categories, Gaps/Missing Data: Complete deletion, Heuristic Method: Nearest-Neighbor-Interchange (NNJ), Branch Swap Filter Strong, Number of Threads 4 (File S17: VP24 Tree MTSX; File S18: VP24 Tree NWK);

E – Clusters/singletons were defined based on tree topology (Cluster/Singleton column);

F – Bootstrap values corresponding to the ancestor of each cluster/singleton were recorded (Bootstrap II column).

VP30 phase (Table S1: Experimental phases, VP30 spreadsheet):

A – Amino acid sequences were manually curated (text editing). The sequence of the M (Matrix) protein NC_001542.1 (*Lyssavirus rabies*) was included as outgroup, and the

following species were excluded: *Thamnovirus kanderense* (KANV), *Thamnovirus percae* (FIWV), *Thamnovirus thamnaconi* (HUJV), and *Oblavirus percae* (OBLV);

B – Alignment was performed using the MUSCLE algorithm (Multiple Sequence Comparison by Log-Expectation) under default settings;

C – Model testing was conducted by Maximum Likelihood (ML) with the following configuration: Automatic (Neighbor-joining tree), Approach Full (Slow), Gaps/Missing Data Complete deletion, Branch Swap Filter Strong, Number of Threads 4 (File S19: Model Testing VP30);

D – Phylogenetic reconstruction was performed with the following parameters: Adaptive Bootstrap (threshold 0.05), Maximum Likelihood method, LG model, Rates among Sites: Gamma Distributed (G) with 5 categories, Gaps/Missing Data: Complete deletion, Heuristic Method: Nearest-Neighbor-Interchange (NNJ), Branch Swap Filter Strong, Number of Threads 4 (File S20: VP30 Tree MTSX; File S21: VP30 Tree NWK);

E – Clusters/singletons were defined based on tree topology (Cluster/Singleton column);

F – Bootstrap values corresponding to the ancestor of each cluster/singleton were recorded (Bootstrap II column).

VP35 phase (Table S1: Experimental phases, VP35 spreadsheet):

A – Amino acid sequences were manually curated (text editing). The sequence of the M (Matrix) protein NC_001542.1 (*Lyssavirus rabies*) was included as outgroup, and the following species were excluded: *Striavirus antennarii* (XILV), *Thamnovirus kanderense* (KANV), *Thamnovirus percae* (FIWV), *Thamnovirus thamnaconi* (HUJV), and *Oblavirus percae* (OBLV);

B – Alignment was performed using the MUSCLE algorithm (Multiple Sequence Comparison by Log-Expectation) under default settings;

C – Model testing was conducted by Maximum Likelihood (ML) with the following configuration: Automatic (Neighbor-joining tree), Approach Full (Slow), Gaps/Missing Data Complete deletion, Branch Swap Filter Strong, Number of Threads 4 (File S22: Model Testing VP35);

D – Phylogenetic reconstruction was performed with the following parameters: Adaptive Bootstrap (threshold 0.05), Maximum Likelihood method, LG model, Rates among Sites: Gamma Distributed (G) with 5 categories, Gaps/Missing Data: Complete deletion, Heuristic Method: Nearest-Neighbor-Interchange (NNJ), Branch Swap Filter Strong, Number of Threads 4 (File S23: VP35 Tree MTSX; File S24: VP35 Tree NWK);

E – Clusters/singletons were defined based on tree topology (Cluster/Singleton column);

F – Bootstrap values corresponding to the ancestor of each cluster/singleton were recorded (Bootstrap II column).

VP40 phase (Table S1: Experimental phases, VP40 spreadsheet):

A – Amino acid sequences were manually curated (text editing). The sequence of the M (Matrix) protein NC_001542.1 (*Lyssavirus rabies*) was included as outgroup, and the following species were excluded: *Thamnovirus kanderense* (KANV), *Thamnovirus percae* (FIWV), *Thamnovirus thamnaconi* (HUJV), and *Oblavirus percae* (OBLV);

B – Alignment was performed using the MUSCLE algorithm (Multiple Sequence Comparison by Log-Expectation) under default settings;

C – Model testing was conducted by Maximum Likelihood (ML) with the following configuration: Automatic (Neighbor-joining tree), Approach Full (Slow), Gaps/Missing Data Complete deletion, Branch Swap Filter Strong, Number of Threads 4 (File S25: Model Testing VP40);

D – Phylogenetic reconstruction was performed with the following parameters: Adaptive Bootstrap (threshold 0.05), Maximum Likelihood method, LG model, Rates among Sites: Gamma Distributed (G) with 5 categories, Gaps/Missing Data: Complete deletion, Heuristic Method: Nearest-Neighbor-Interchange (NNJ), Branch Swap Filter Strong, Number of Threads 4 (File S26: VP40 Tree MTSX; File S27: VP40 Tree NWK);

E – Clusters/singletons were defined based on tree topology (Cluster/Singleton column);

F – Bootstrap values corresponding to the ancestor of each cluster/singleton were recorded (Bootstrap II column).
