# Supplementary figures and images for: Exploratory Temporal and Evolutionary Insights into the Filoviridae Family Through Multiprotein Phylogeny
Source: Microorganisms. 2025 Oct 17;13(10):2388. doi: 10.3390/microorganisms13102388 (PMC12566026; doi:10.3390/microorganisms13102388)

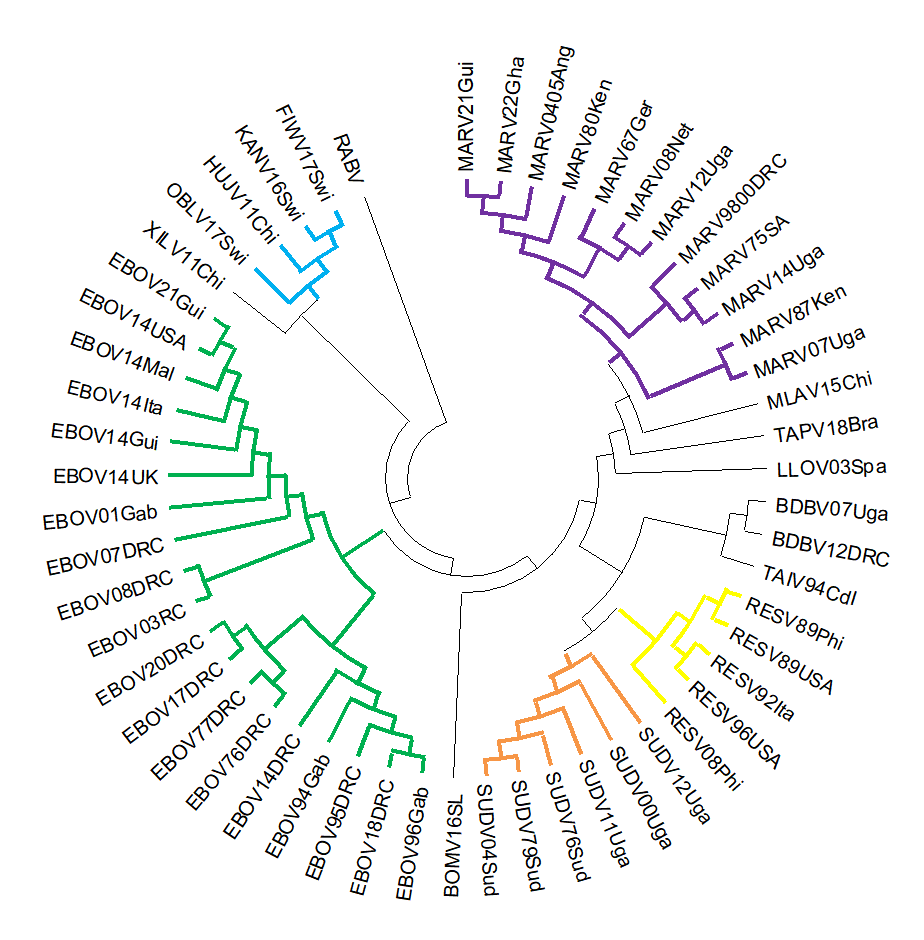

Supplement: Supplementary file 1 [file microorganisms-13-02388-s001.zip › Figure S1 Filoviral Phylogenetic Tree (GP).png]

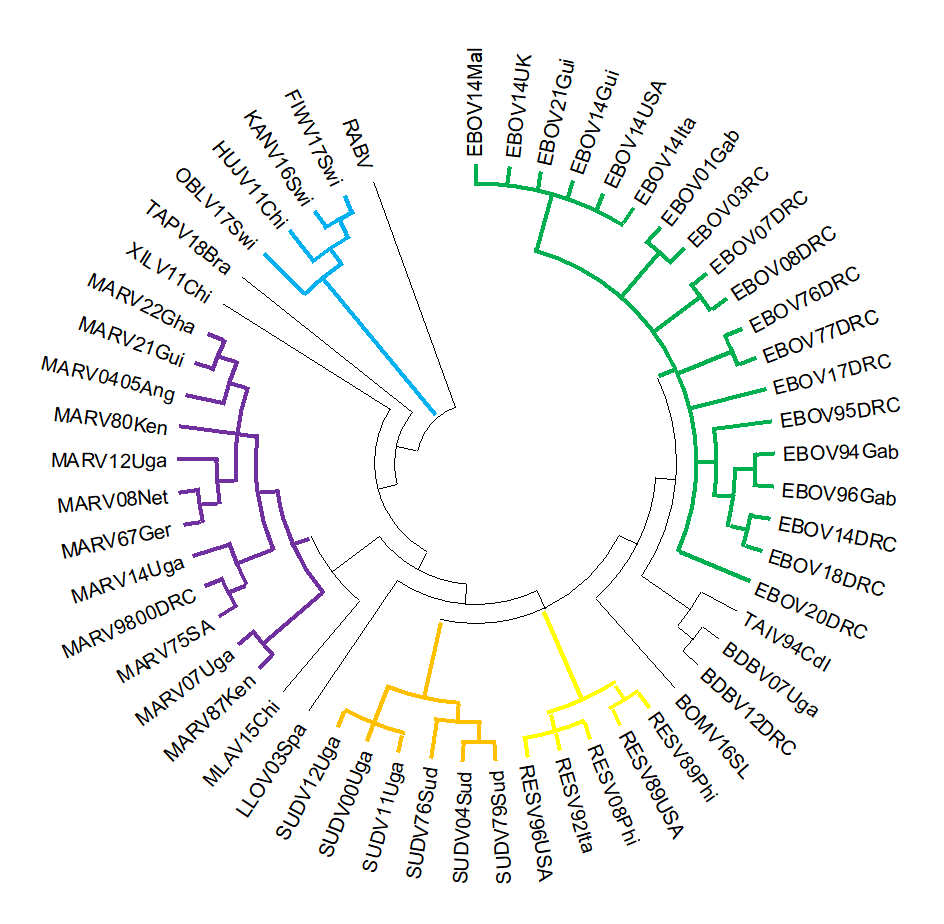

Supplement: Supplementary file 1 [file microorganisms-13-02388-s001.zip › Figure S2 Filoviral Phylogenetic Tree (L).png]

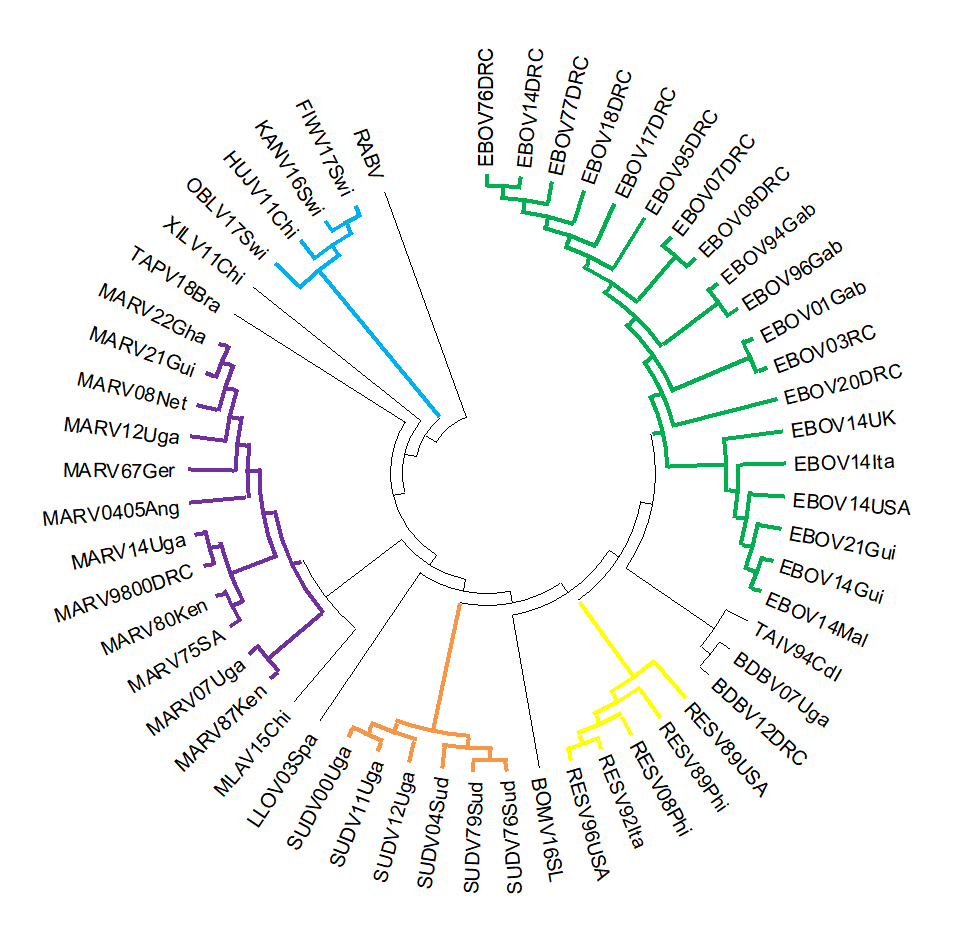

Supplement: Supplementary file 1 [file microorganisms-13-02388-s001.zip › Figure S3 Filoviral Phylogenetic Tree (NP).png]

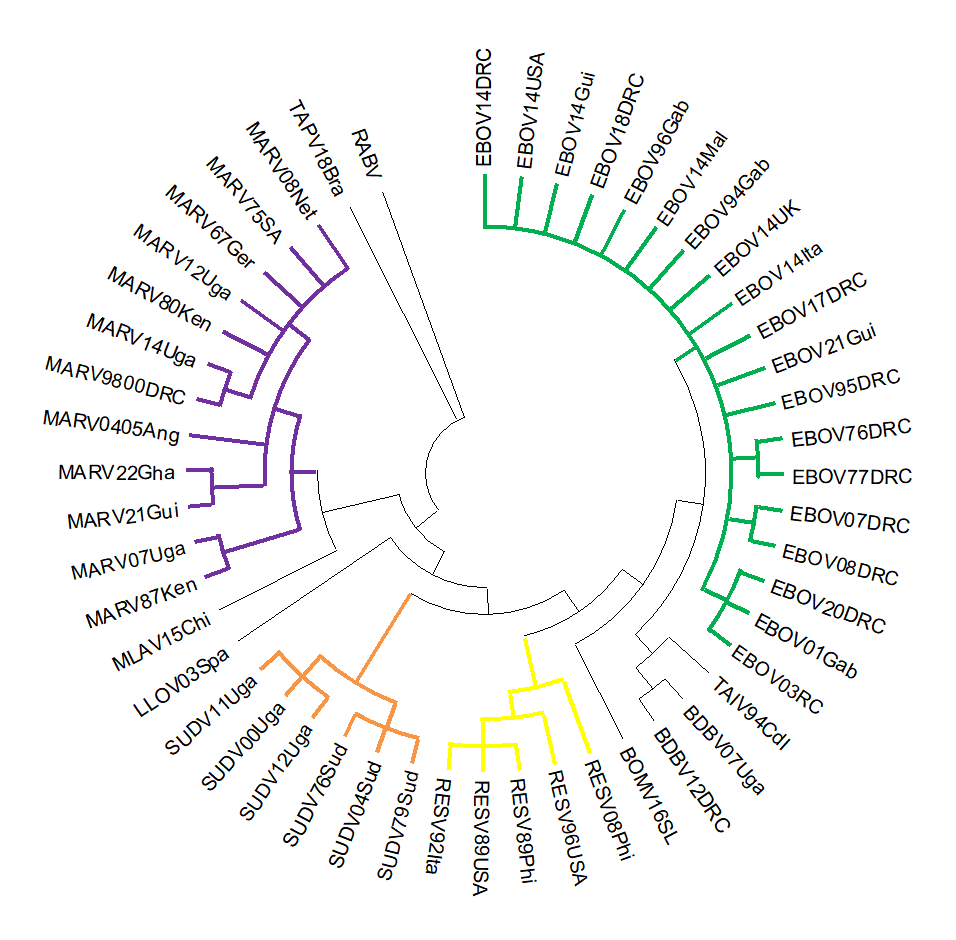

Supplement: Supplementary file 1 [file microorganisms-13-02388-s001.zip › Figure S4 Filoviral Phylogenetic Tree (VP24).png]

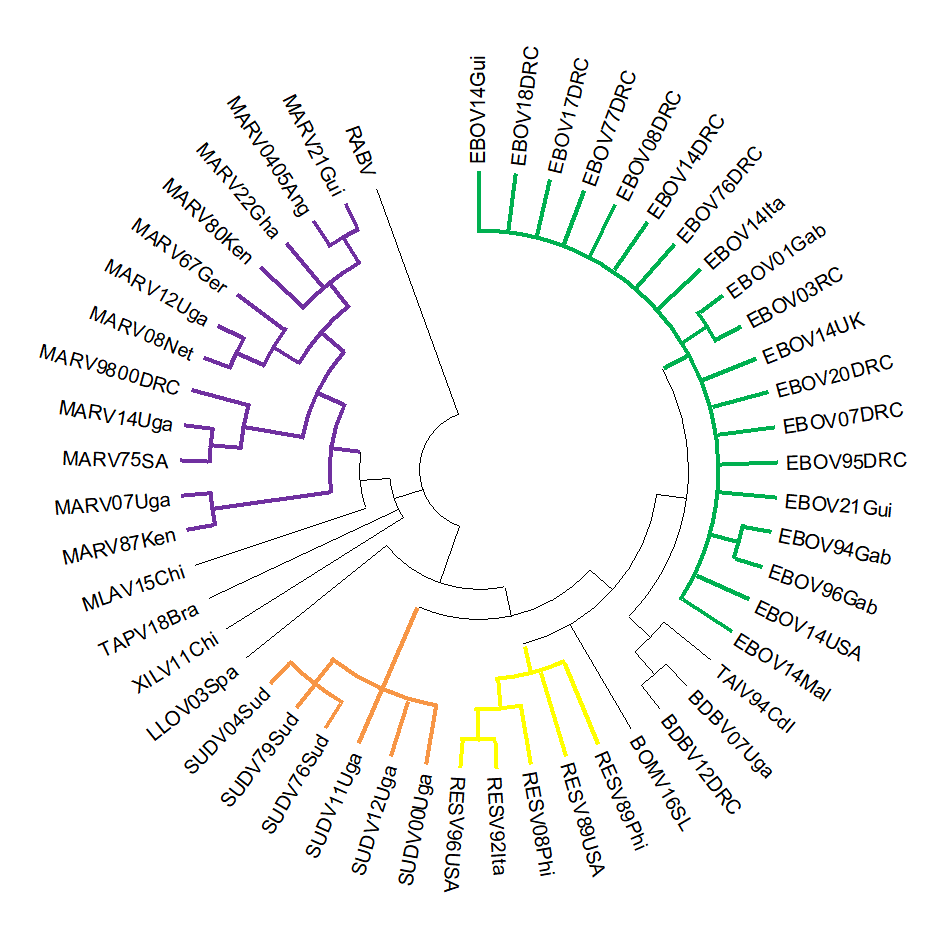

Supplement: Supplementary file 1 [file microorganisms-13-02388-s001.zip › Figure S5 Filoviral Phylogenetic Tree (VP30).png]

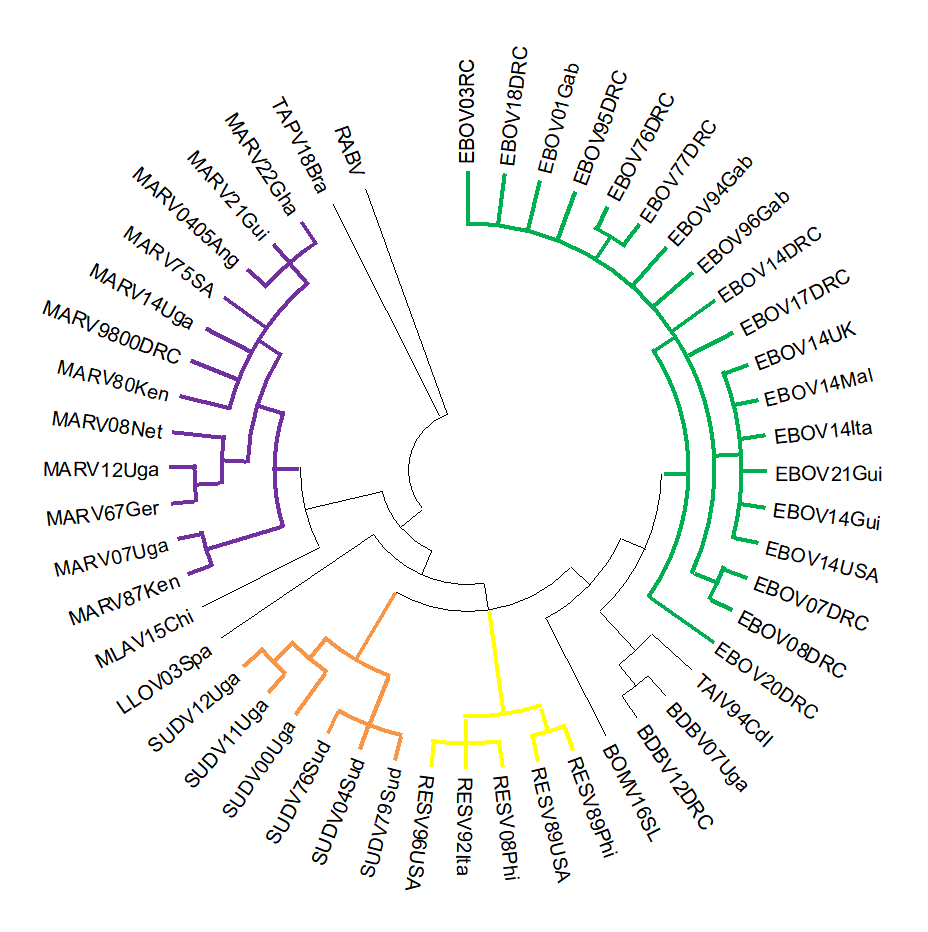

Supplement: Supplementary file 1 [file microorganisms-13-02388-s001.zip › Figure S6 Filoviral Phylogenetic Tree (VP35).png]

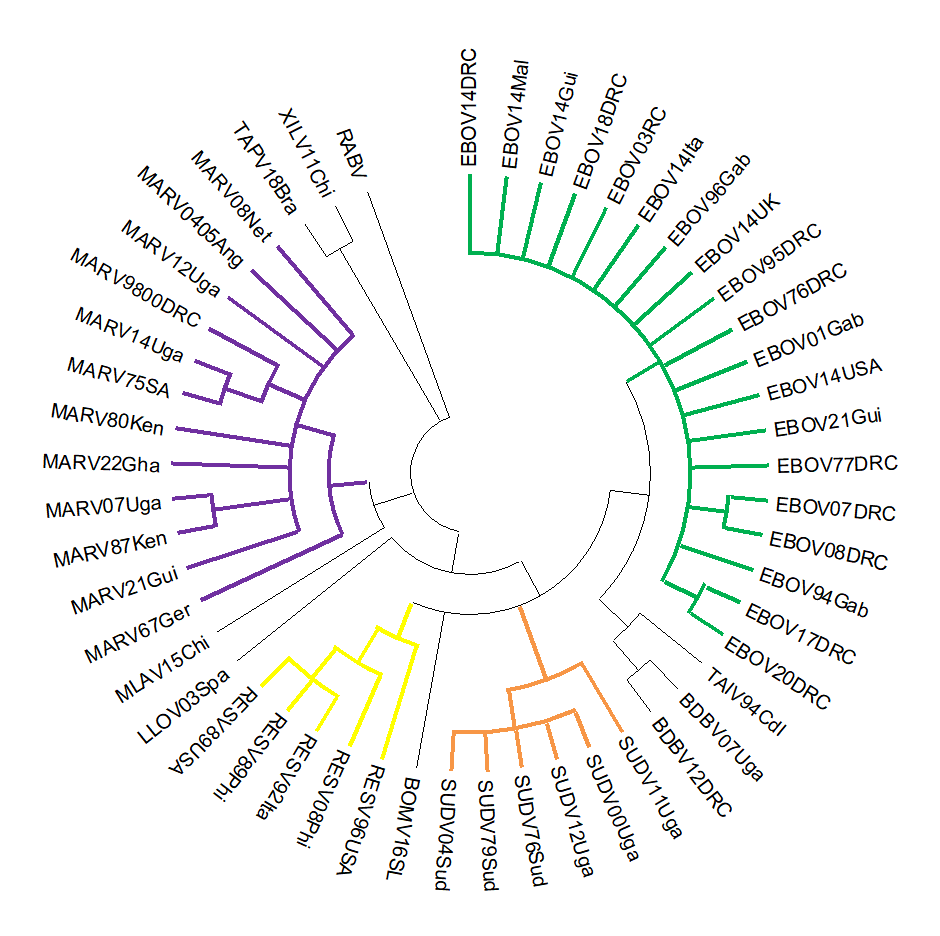

Supplement: Supplementary file 1 [file microorganisms-13-02388-s001.zip › Figure S7 Filoviral Phylogenetic Tree (VP40).png]
